# Clicking in harmony: exploring the bio-orthogonal overlap in click chemistry

**DOI:** 10.1039/d4ra00494a

**Published:** 2024-03-01

**Authors:** Mehak, Gurleen Singh, Riddima Singh, Gurjaspreet Singh, Jigmat Stanzin, Harminder Singh, Gurpreet Kaur, Jandeep Singh

**Affiliations:** a School of Chemical Engineering and Physical Sciences, Lovely Professional University Phagwara-144411 Punjab India singhjandeep@gmail.com; b Department of Chemistry and Centre of Advanced Studies in Chemistry, Panjab University Chandigarh-160014 India; c Department of Chemistry, Gujranwala Guru Nanak Khalsa College Civil Lines Ludhiana–141001 Punjab India

## Abstract

In the quest to scrutinize and modify biological systems, the global research community has continued to explore bio-orthogonal click reactions, a set of reactions exclusively targeting non-native molecules within biological systems. These methodologies have brought about a paradigm shift, demonstrating the feasibility of artificial chemical reactions occurring on cellular surfaces, in the cell cytosol, or within the body – an accomplishment challenging to achieve with the majority of conventional chemical reactions. This review delves into the principles of bio-orthogonal click chemistry, contrasting metal-catalyzed and metal-free reactions of bio-orthogonal nature. It comprehensively explores mechanistic details and applications, highlighting the versatility and potential of this methodology in diverse scientific contexts, from cell labelling to biosensing and polymer synthesis. Researchers globally continue to advance this powerful tool for precise and selective manipulation of biomolecules in complex biological systems.

## Abstract 2

The fundamentals of bio-orthogonal click chemistry are investigated, while exploring mechanistic intricacies, demonstrating the adaptability and promise of this methodology.

## Graphical abstract

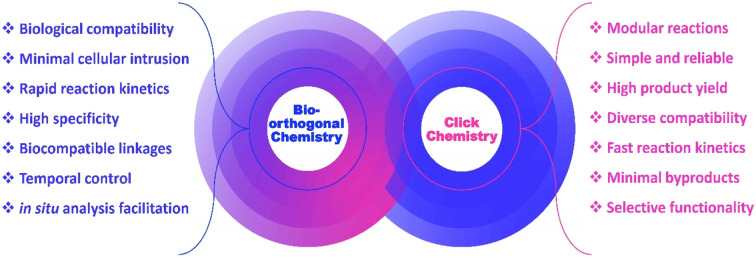

## 1. Introduction

**Fig. 1:**
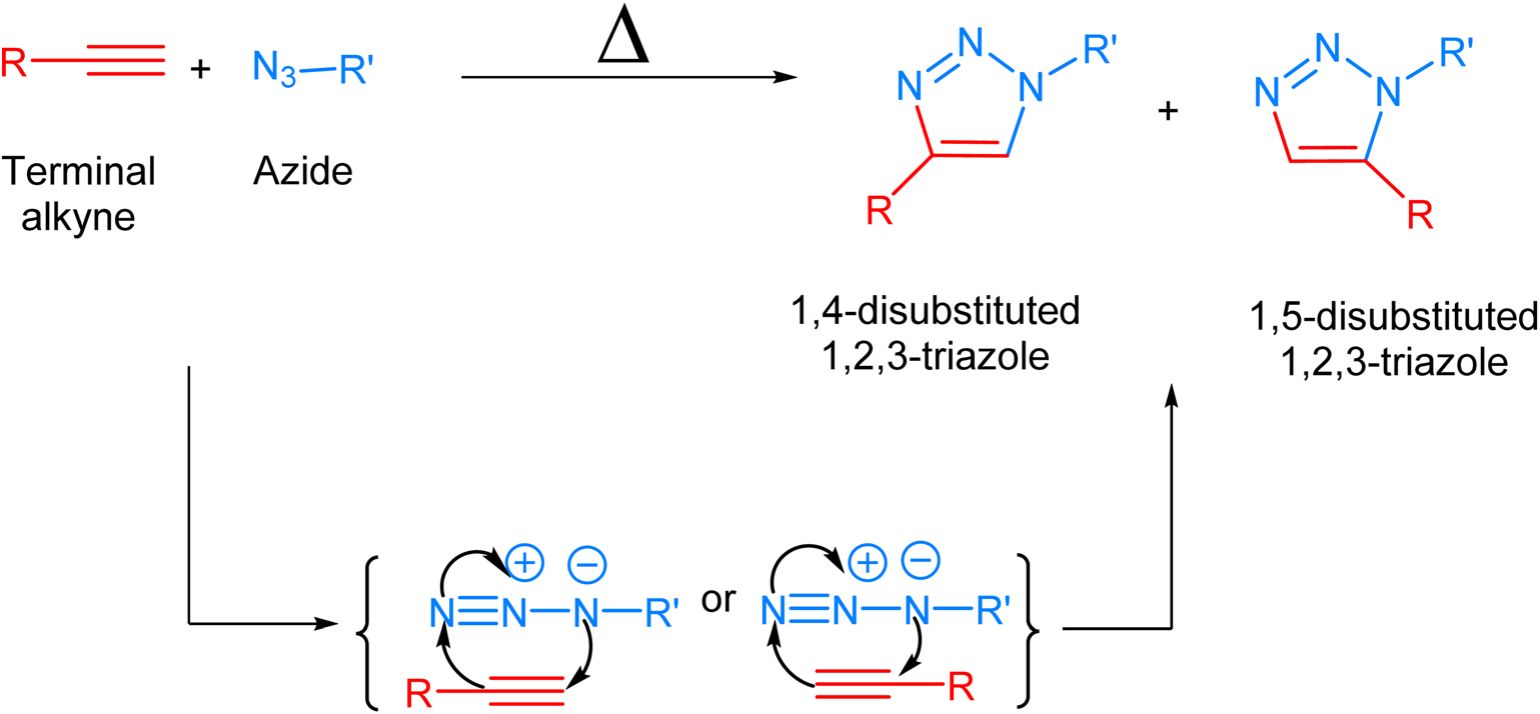
1,3-Dipolar cycloaddition between terminal alkyne and azide giving isomeric mixture of 1,4- and 1,5-disubstituted 1,2,3-triazole.

Biological systems are accompanied by delicate networks of native biomolecules, vital for conventional functioning. For scrutinizing and modifying the biological systems, scientists have introduced a fresh set of reactions, namely bio-orthogonal click reactions, which exclusively alter non-native target molecules in the biological system, heretofore introduced, making it an ideal reaction for employing *in vitro*.^1–3^ Bio-orthogonal chemistry enables the execution of organic synthesis typically conducted in a laboratory within living organisms and cells.^4^ In contrast to numerous laboratory reactions, bio-orthogonal reactions are not designed for the bulk production of materials. Rather, their purpose is to covalently modify biomolecules with non-native functional groups under biological conditions, facilitating their examination and manipulation.^5,6^ The roots of bio-orthogonal chemistry can be traced way back to the Huisgen 1,3-dipolar cycloaddition of organic azide and terminal alkyne to yield a racemic mixture of 1,4-disubstituted and 1,5-disubstituted 1,2,3-triazoles (Fig. 1).^7^ The versatility of this reaction for organic synthesis and in pharmaceutical science owes to the fact that the reaction was resourceful and comes up with a ‘linker’ compound that remains unaltered in the standard reactions.^8^ Although the methodology has many ascendancies, it is also associated with long reaction kinetics, harsh reaction conditions, non-regioselectivity (1,4 and 1,5-disubstituted), and trouble in column chromatography.^9^

**Fig. 2:**
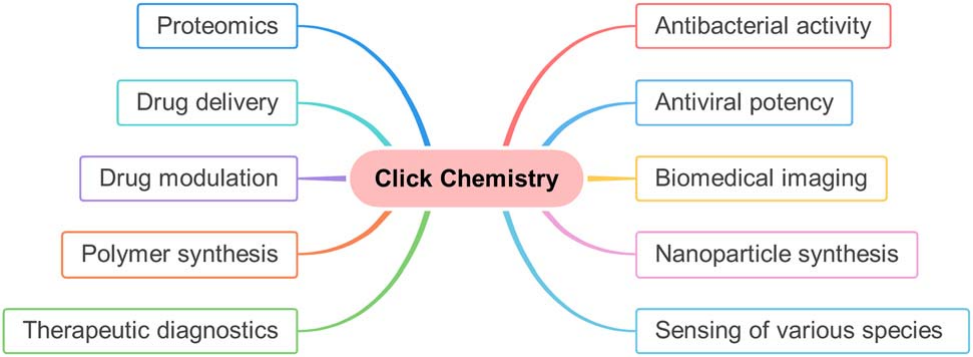
An illustration of the diverse applications of ‘Click Chemistry’.

An innovative chemist, Dr Karl B. Sharpless reported the immensely enhanced reaction rate (of the order 10^7^ times) of Huisgen cycloaddition with the regioselective synthesis of 1,4-disubstituted 1,2,3-triazole as the sole product in high-to-excellent yield as compared to traditional reaction on utilizing Cu(i) as a catalyst.^10^ Similar work was also reported independently by Dr Morten M. Meldal, for which both the eminent researchers were awarded the Noble Prize in Chemistry 2022. According to Sharpless and his co-worker,^10^ the driving force for this irreversible reaction is thermodynamically provided (usually more than 20 kcal mol^−1^) and the synthesized compound prepared could be readily segregated by non-chromatographic techniques.^11,12^ This robust and efficient synthetic methodology, characterized by fast kinetics, no by-product formation, benign conditions, high chemoselectivity and regioselectivity, was named ‘Click Chemistry’ by Sharpless *et al.*^10^ The reaction pathways sharing the aforementioned characteristics as well as trajectories have significance in diverse research fields^13,14^ such as in boosting the quality of the compound library, drug delivery,^15,16^ drug modulation,^17^ polymer synthesis,^18^ therapeutic diagnostics,^19,20^ proteomics,^21^ nanoparticles synthesis,^22–24^ detection of different species^25–27^ and are also evaluated as substantial routes for producing radiopharmaceutical agents which are employed in biomedical imaging techniques such as PET/CT and MRI.^28–31^ Moreover, these click reactions have been employed in the production of antiviral medications designed to combat viral infections, including but not limited to COVID-19, HIV, influenza, and various other viral strains, demonstrating their remarkable efficacy in recent times^32–35^ (Fig. 2).

**Fig. 3:**
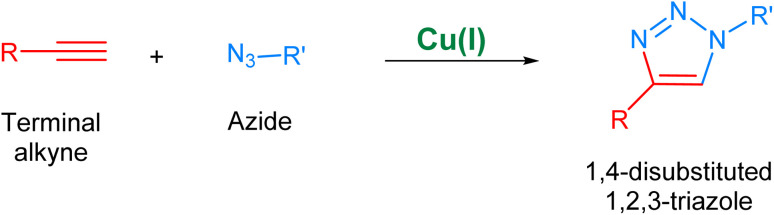
An illustration of the Cu(i)-catalyzed alkyne–azide cycloaddition (CuAAC) leading to the regioselective synthesis of 1,4-disubstituted 1,2,3-triazole.

The Cu(i) catalyzed alkyne–azide cycloaddition, *i.e.*, CuAAC^36^ (Fig. 3), considered as the archetypal reaction of ‘Click Chemistry’, is rendered as unsusceptible to the electronic effects of substituents of the two reactants present and can take place in a variety of solvents without any organic co-solvent.^37,38^ Additionally, the regioselectivity of this methodology owing to the implementation of Cu(i) catalyst further adds to its desirability. Nonetheless, the utilization of CuAAC in reactions conducted under physiological conditions is associated with the issue of copper toxicity, which is attributed to the inherent pro-oxidant nature of copper, which results in the generation of free radicals within living organisms which consequently inflict damage to cellular structures and DNA, culminating in inflammatory responses and the development of chronic diseases.^39,40^

**Fig. 4:**
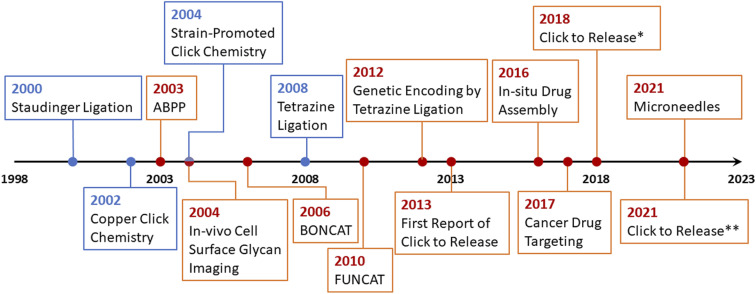
An illustration of the timeline of the progressive developments and substantial applications of bio-orthogonal click chemistry.^6^

To overcome the issue of metal toxicity, an American chemist Dr Carolyn R. Bertozzi and her co-workers in 2004 introduced ‘Bio-orthogonal Click Chemistry’ which is essentially a Cu-free version of the click reaction involving strained cycloalkynes, and serves all the criteria of biocompatible reactions *i.e.*, no toxic by-products formation, non-interference with normal physiological functions and most importantly does not trigger any immune response.^42^ Bio-orthogonal click reactions are vast and include a wide range of applications with characteristic features of excellent regioselectivity and chemoselectivity, high to excellent yield accompanied by no by-products, and fast reaction kinetics. The reactive moieties can artificially be introduced onto the surface of a substrate which consequently makes them selectively reactive towards the counterpart making bio-orthogonal bonds under mild physiological conditions.^41,42^ Adequacy of this phenomenon enables the addition of a preferred compound to target biomolecule using a cell's metabolic mechanism and this standout strategy serves in detecting carcinomas,^43^ synthesis of thermosets material having characteristic properties, and sustainability.^44^ Furthermore, it is also utilized globally for the fabrication of ultrasensitive biosensors for the detection of microRNAs and DNA,^45,46^ labelling^47,48^ and *in vivo* applications.^49–51^ For this pioneering work, Dr Bertozzi was also conferred with the Nobel prize in Chemistry 2022 together with Dr Sharpless and Dr Meldal. In 2021, Bird *et al.* presented an accurate timeline of the significant events in the development of bio-orthogonal chemistry and its substantial applications,^6^ which is represented in Fig. 4.

The constructiveness of the ‘bio-orthogonal click’ phenomenon makes it a powerful tool in the medical and synthetic field, enabling precise and selective labelling, imaging, covalent cell engineering and manipulation of biomolecules in complex biological systems. Numerous researchers worldwide are exploring the advances in the click reaction, thereby enhancing its applicability in both biological and chemical domains.^52–56^ The present study elucidates the principles underlying bio-orthogonal click chemistry, emphasizing the fundamental distinctions between conventional metal-catalyzed and metal-free bio-orthogonal click reactions. The diverse approaches encompassed under bio-orthogonal click reactions and their mechanistic details are comprehensively discussed, shedding light on their advantageous features. The application of this methodology spans across a broad spectrum of research endeavours, encompassing areas such as cell labelling, biosensing, polymer synthesis, *etc.* A detailed exposition is provided for the same, underscoring the demonstrated utility and potential of this reaction methodology within diverse scientific contexts.

## 2. Types of bio-orthogonal click reactions: thrust and mechanistic aspects

Bio-orthogonal click chemistry refers to a set of chemical reactions that can occur selectively in biological systems without interfering with native biochemical processes. In order to qualify as bio-orthogonal, a chemical reaction must adhere to a set of stringent criteria. Firstly, the reaction should transpire under conditions similar to the prevailing temperatures and pH levels within the physiological environment.^57^ Secondly, it must exhibit selectivity in yielding products while remaining resistant to water and inherent biological molecules possessing nucleophilic, electrophilic, reductive, or oxidative properties typically encountered in intricate biological surroundings.^58^ Furthermore, the bio-orthogonal reaction must possess rapid kinetics, even at low concentrations.^3^ Lastly, the involvement of functional groups not naturally occurring within biological systems is an essential parameter for such reactions.^59^ Several reaction methodologies are encompassed under bio-orthogonal click reactions, including but not limited to those mentioned below, with their specific advantages and limitations compiled in Table 1.

**Table 1:** The distinct bio-orthogonal ‘click’ reactions with their advantages and limitations

Type of click reaction	Strength	Weakness	Ref.
SPAAC	• No external catalyst required	• Relatively slower reaction kinetics	61 and 80
	• Precisely selective		
	• Biocompatible	• Possibility of cyclooctyne interaction with nucleophiles in living systems	
	• Inert towards physiological environment		
Staudinger ligation	• Highly selective approach for chemical labelling *in vivo*	• Low stability of iminophosphoranes/azaylides	64 and 81
	• Synthesis of a library of bioconjugates implemented in pharmaceutical research	• Vulnerability of phosphorous compounds towards moisture and/or air	
Thiol–ene reaction	• Homogenous polymer network *via* regulated step-growth and chain-growth processes	• Oxygen inhibition, complex volume relaxation, and stress buildup	67 and 68
	• Rapid and uniform synthesis of thiol–ene networks under ambient air conditions		
IEDDA	• No coupling reagent or catalyst required	• Sensitivity of *trans*-cyclooctene and 1,2,4,5-tetrazine to acids, thiols, copper ions, and bases	71 and 82
	• Fast reaction kinetics in aqueous environment		
	• Better kinetics when compared with the conventional DA reaction	• Difficult to investigate the reaction *in vivo* with smaller biomolecules, such as peptides	
	• Enhanced stability of reaction owing to electron-withdrawing groups attached with tetrazine reagent	• Adopting such an approach at a clinical level presents a logistical difficulty	
	• Water, biological media and organic solvents can all be used for the reaction		
Tetrazole ligation	• Reactivity-based tool in biological systems owing to the biocompatible light source activation and genetically encodable alkene reporters	• Limited biological relevance of reactions initiated by UV light in the range of 300–360 nm owing to phytotoxicity of the radiations to the living cells	74 and 83
	• Precise control over both spatial and temporal aspects owing to activation only in the presence of specific light wavelengths		
Oxime ligation	• Reaction proceeds under mild acidic conditions in aqueous systems	• Hydrolytic instability of oximes, like other condensation products such as imines and hydrazones in aqueous media	77
	• Compatible with the majority of biomolecule functionalities	• Difficult synthesis and storage of aldehyde or amino-oxy functionalized biomolecules	
	• The only side-product formed is water	• Susceptibility of aldehydes to spontaneous oxidation or self-coupling	

### 2.1. Strain-promoted azide–alkyne cycloaddition (SPAAC)

**Fig. 5:**
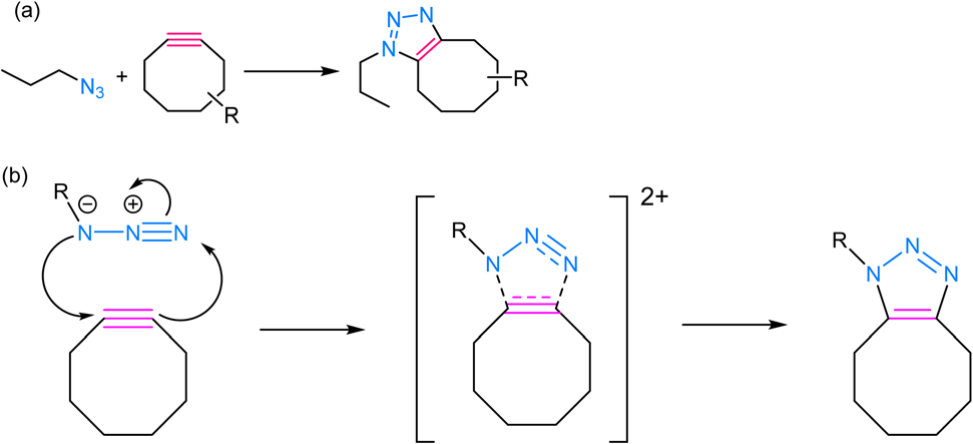
(a) An illustration of strained-promoted alkyne–azide cycloaddition; (b) reaction mechanism depicting the formation of a 1,2,3-triazole ring by the cycloaddition of an alkyne and an azide.

SPAAC is a powerful and widely used click chemistry reaction that plays a crucial role in chemical biology and materials science and involves the reaction between an azide and an alkyne, resulting in the formation of a stable 1,2,3-triazole linkage (Fig. 5). Unlike the traditional CuAAC, SPAAC is bio-orthogonal, *i.e.*, it can occur in the presence of biological systems without interfering with native biological processes.^60,61^ The term “strain-promoted” emphasizes the requirement for high ring strain in the reactants, typically involving cyclooctynes, to facilitate the reaction. The strain energy facilitates the formation of a reactive intermediate, which rapidly reacts with azides to yield triazole products, thereby rendering SPAAC substantial in the context of bioconjugation and biomolecule labelling, as it avoids the potential harm associated with metal catalysts and ensures minimal interference with biological systems.^62,63^ Furthermore, SPAAC's metal-free nature simplifies purification and reduces the risk of metal contamination in the final products, making it a safer and more versatile choice for a wide range of applications.

### 2.2. Staudinger ligation

**Fig. 6:**
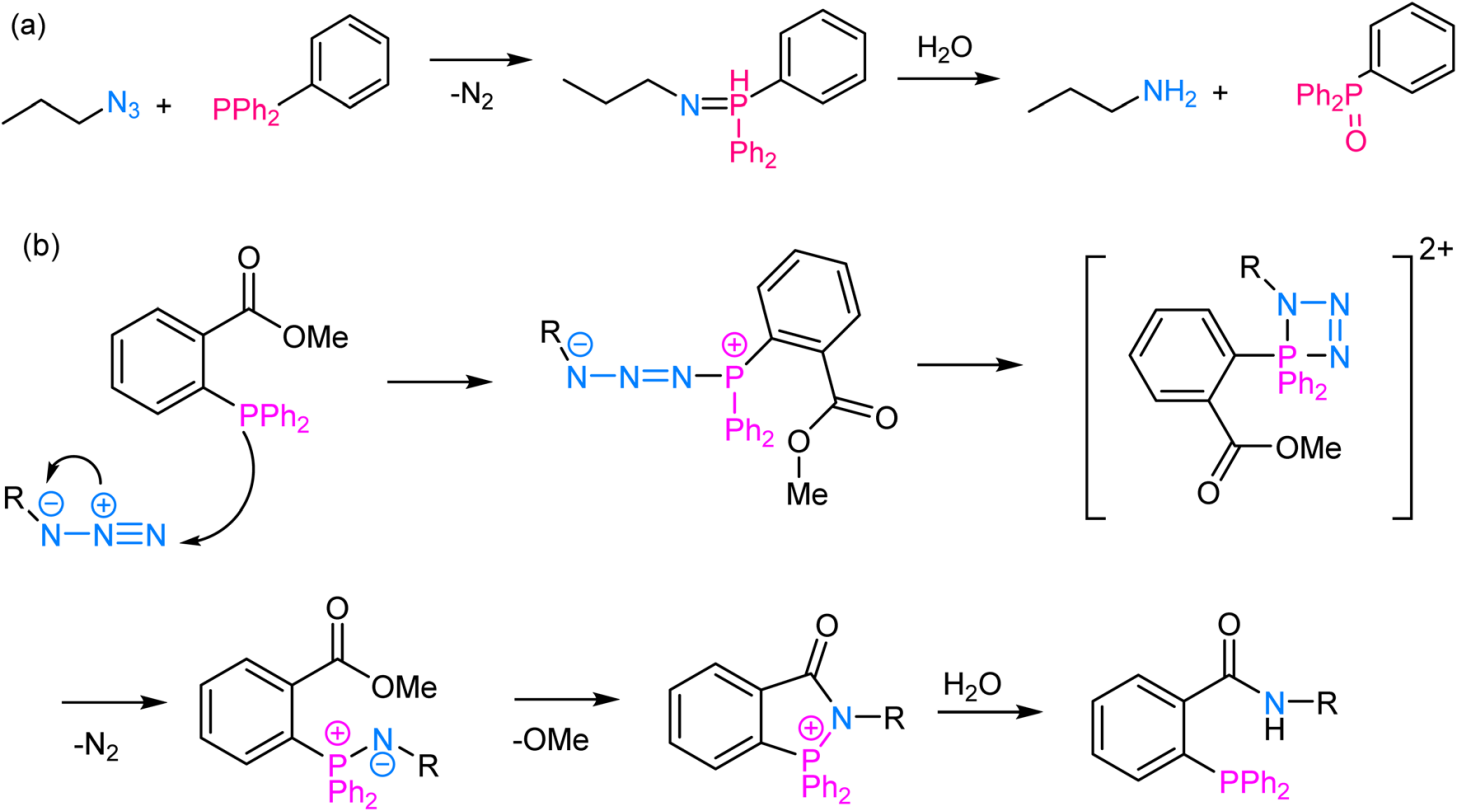
(a) An illustration of Staudinger ligation; (b) reaction mechanism depicting the nucleophilic attack of a phosphine group on an azide moiety, thereby enabling Staudinger ligation.

The Staudinger ligation involves the reaction between an azide and a phosphine wherein the reaction proceeds through a phosphine oxide intermediate and is widely used for labelling biomolecules. At its core, the reaction involves the nucleophilic attack of a phosphine (typically triarylphosphine) on an azide group, leading to the formation of an iminophosphorane intermediate, which subsequently undergoes rearrangement to yield an amine and a phosphine oxide as final products^64,65^ (Fig. 6). The reaction proceeds efficiently under mild physiological conditions, making it suitable for use in living systems. One of the key advantages of Staudinger ligation is its selectivity, as the azide group is virtually absent in biological molecules, and the triarylphosphine is unreactive toward most other functional groups, thereby ensuring minimal interference with the cellular processes.^66^ This selectivity allows for precise and targeted modifications of biomolecules, such as labelling specific proteins with fluorophores or other tags, tracking their localization, and probing their interactions with other molecules in a non-invasive manner.

### 2.3. Thiol–ene click chemistry

**Fig. 7:**
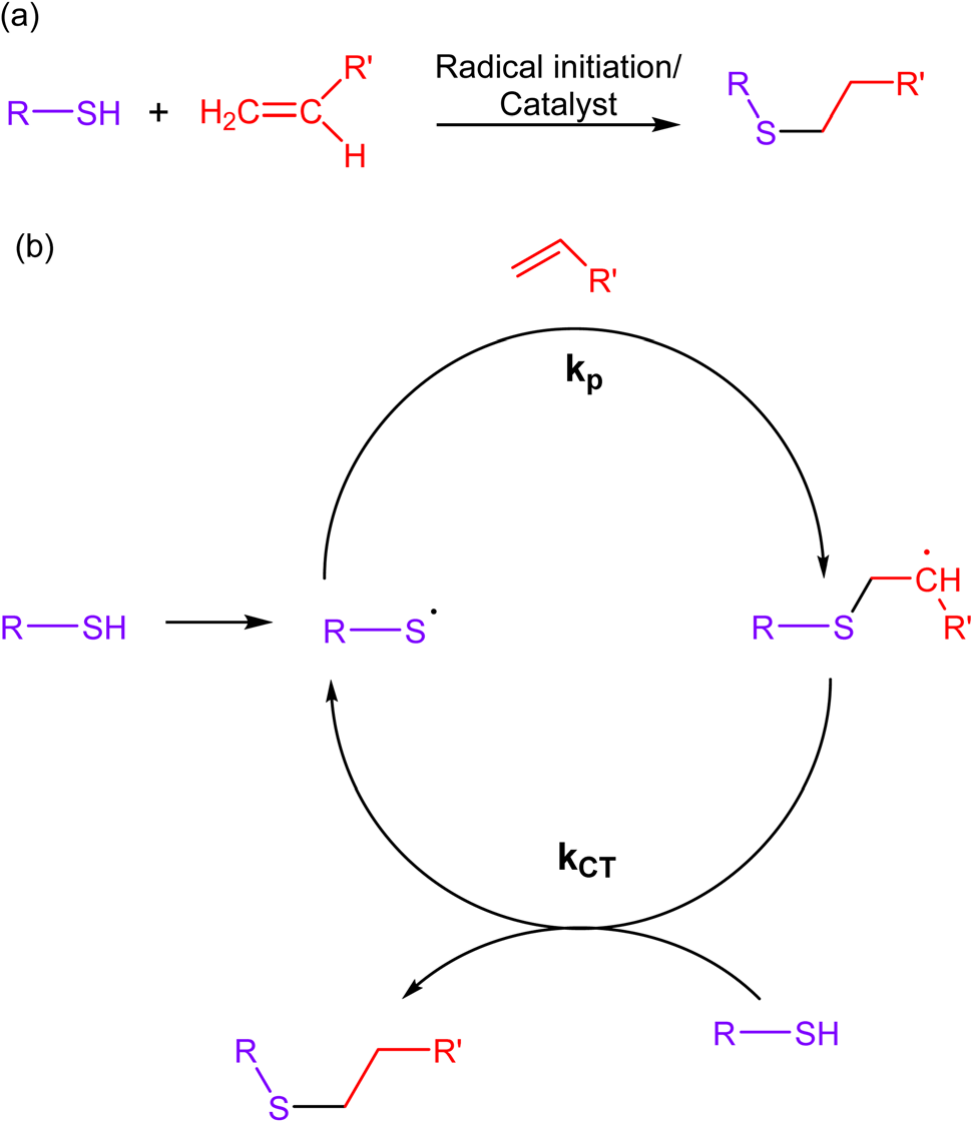
(a) An illustration of thiol–ene click reaction; (b) reaction mechanism depicting the progress of the reaction *via* free radical mechanism, giving a composite product.

Thiol–ene reactions involve the reaction of thiols (sulfhydryl groups) with alkenes, wherein a thiol and an alkene (ene) react through a radical-mediated process, forming a carbon–sulfur bond as a result. The mechanistic approach of this reaction involves the initiation of a radical chain reaction through a photochemical or chemical stimulus, leading to the selective formation of a thioether bond (Fig. 7). Additionally, the scarcity of thiol and alkene functional groups in biological molecules ensures minimal cross-reactivity with other chemical moieties present in living systems.^67–69^

### 2.4. Inverse electron demand Diels–Alder reaction/tetrazine ligation

**Fig. 8:**
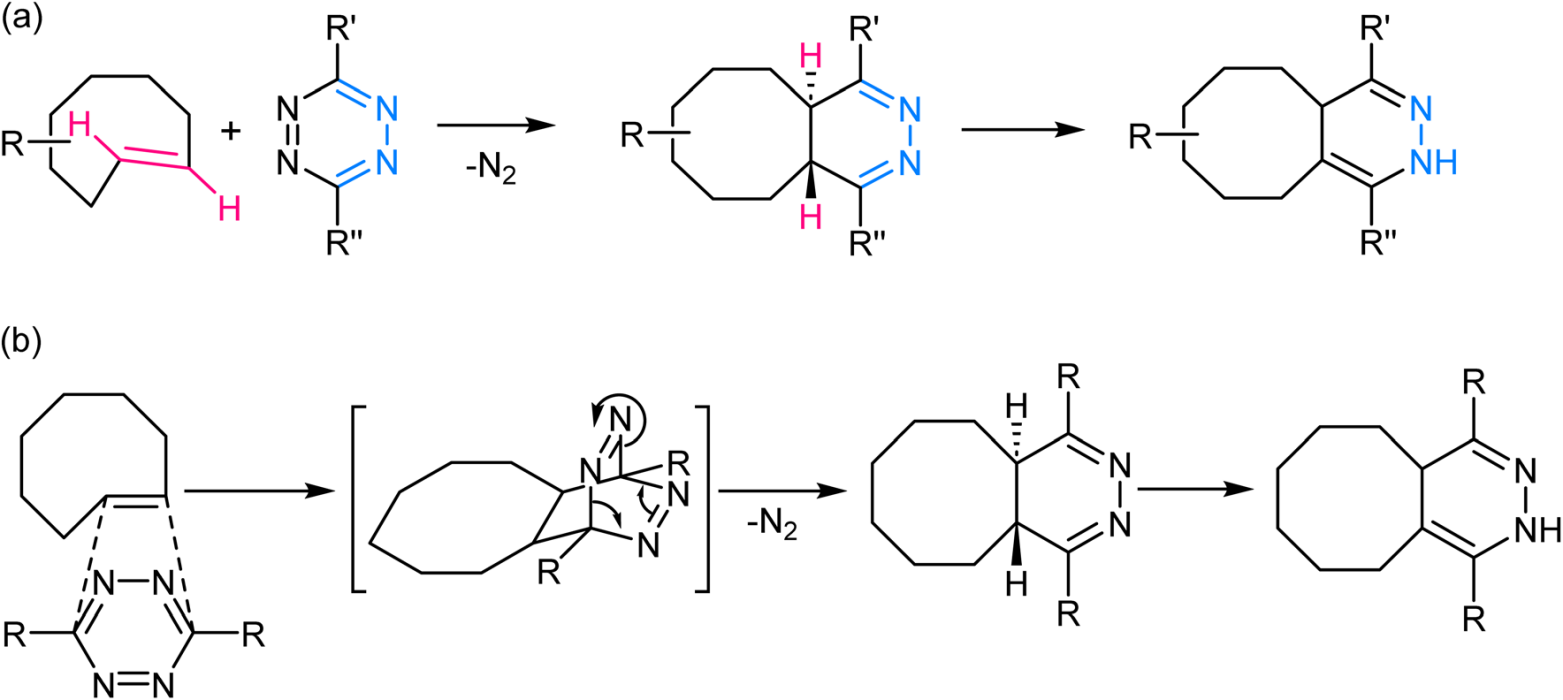
(a) An illustration of inverse electron demand Diels–Alder reaction; (b) reaction mechanism exhibiting the affinity of the dienophile towards electron-rich tetrazine ring.

This reaction involves the highly selective and rapid reaction between a tetrazine and an electron-deficient dienophile, often a *trans*-cyclooctene or norbornene derivative to yield a fused dihydropyridazine product (Fig. 8). The reaction is kinetically favoured due to the inherent strain in the tetrazine ring, which is relieved upon formation of the cycloadduct. The rapid kinetics of this reaction, often occurring within seconds, further contributes to its effectiveness in various *in vivo* and *in vitro* contexts.^70–72^

### 2.5. Tetrazole ligation

**Fig. 9:**
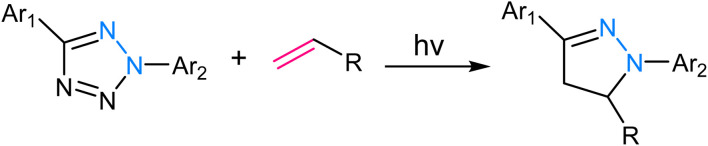
An illustration of tetrazole ligation.

Tetrazole ligation relies on the unique reactivity of tetrazole derivatives under specific photoirradiation conditions. This reaction involves the light-triggered formation of covalent bonds between tetrazoles and strained alkenes, typically bicyclo[6.1.0]nonyne (BCN) or *trans*-cyclooctene (TCO) (Fig. 9). The approach provides spatiotemporal control, as it only proceeds when exposed to specific wavelengths of light, allowing for precise manipulation of molecular interactions in complex biological systems.^73,74^ The mechanism of tetrazole ligation is based on the rapid, orthogonal reaction between tetrazole and the dienophile upon exposure to light, forming a stable triazole bond. The bio-orthogonality of this reaction is maintained due to the scarcity of tetrazole and dienophile groups in biological molecules, minimizing potential cross-reactivity. This control over reaction initiation has enabled applications in live-cell imaging, targeted drug delivery, and the selective labelling of proteins or other biomolecules.^75,76^

### 2.6. Oxime ligation

**Fig. 10:**
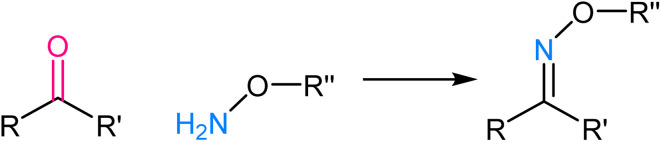
An illustration of oxime ligation.

Oxime ligation involves the formation of an oxime bond between an aldehyde or ketone and an aminooxy functional group, such as hydroxylamine or aminooxy biotin. The mechanistic basis for oxime ligation is the nucleophilic attack of the aminooxy group on the carbonyl moiety of the aldehyde or ketone, leading to the stable oxime linkage^77^ (Fig. 10). This reaction is thermodynamically favourable and proceeds under mild, biocompatible conditions, making it well-suited for applications in living systems. The selectivity of oxime ligation is rooted in the rarity of aldehyde and ketone groups in biological molecules, ensuring minimal interference with native cellular processes.^78,79^ As a result, the reaction has found extensive use in biomolecule labelling, bioconjugation, and drug delivery, offering a versatile and reliable method for attaching various functional groups to specific biological targets.

## 3. Advantages of bio-orthogonal click chemistry

Bio-orthogonal click reactions offer several advantages over conventional organic reactions, particularly in chemical biology and biochemistry, as these reactions can occur in the presence of various biomolecules, such as proteins, nucleic acids, and lipids, without interfering with the native biochemical processes of living organisms. The selectivity and orthogonality of these reactions also enables their sequential or simultaneous use, facilitating the design of sophisticated multi-step processes for imaging, drug delivery, and other applications.^84–86^ Furthermore, bio-orthogonal click reactions contribute to the development of bioconjugation strategies for linking synthetic entities to biomolecules with precision, paving the way for innovative advances in diagnostics, therapeutics, and the study of biological processes.^87,88^ Some key advantageous characteristics of bio-orthogonal click reactions are discussed as under:

### 3.1. Bio-orthogonality

Bio-orthogonality refers to the property of a chemical reaction or process that allows it to occur selectively in a specific biological context without interfering with other concurrent biochemical processes. Bio-orthogonal click chemistry is fundamentally designed to be compatible with living systems; it does not interfere with the intricate biochemical processes that occur within cells and therefore can be applied *in vivo*, for tracking and manipulating biological processes. This is challenging to achieve with conventional organic reactions, which are often not compatible with living systems.^89–91^

### 3.2. Non-cytotoxicity

Bio-orthogonal click chemistry typically employs catalysts and reagents that are non-toxic to cells and biomolecules, thereby overcoming the limitation of generating reactive oxygen species (ROS). This reduces the risk of harming living systems and minimizes the potential for unintended side effects.^88,92^

### 3.3. Reaction kinetics

Some bio-orthogonal reactions, such as strain-promoted azide–alkyne cycloaddition (SPAAC) and inverse electron-demand Diels–Alder (IEDDA) reactions, offer fast reaction kinetics. This rapidity is advantageous when conducting experiments that require quick labelling, tagging, or modification of biomolecules.^93,94^

### 3.4. Functional group compatibility

Bio-orthogonal reactions are compatible with a broad range of functional groups, including azides, alkynes, strained cyclooctynes, and more. This versatility allows researchers to modify or conjugate diverse biomolecules, proteins, nucleic acids, and chemical structures.^95,96^

### 3.5. Biomolecule compatibility

Bio-orthogonal click chemistry is designed to work with biomolecules without significantly altering their structure or function. Consequently, the reactions are less likely to interact with or modify other cellular components, leading to more precise and specific labelling and conjugation. This makes it ideal for labelling and modifying proteins, nucleic acids, lipids, and other biologically relevant molecules.^96,97^

## 4. Metal-catalyzed and metal-free bio-orthogonal click reactions: optimizing the bio-orthogonality

Metal-catalyzed and metal-free bio-orthogonal click reactions represent two distinct approaches in bioconjugation chemistry, each offering unique advantages and limitations. In the context of metal-catalyzed click reactions, the challenge lies in achieving this bio-orthogonality while using metal ions as catalysts.^93,98^ Despite their impressive reactivity and efficiency, metal-catalyzed reactions sometimes exhibit limited bio-orthogonality due to the potential for metal-induced cytotoxicity and undesired interactions with biomolecules. This necessitates meticulous optimization of reaction conditions and the development of bio-orthogonal ligands to enhance selectivity.^99,100^

Conversely, metal-free click reactions, being inherently devoid of metal catalysts, tend to offer superior bio-orthogonality. Their compatibility with biological systems is often higher, as they avoid the issues associated with metal-mediated interference. The absence of metal catalysts reduces the risk of cytotoxicity, and these reactions can be fine-tuned to achieve high selectivity.^99,101^ However, metal-free reactions may require longer reaction times or specific conditions, which can influence their practical utility in some applications (Table 2). Balancing the advantages of bio-orthogonality and reaction efficiency is a central consideration when choosing between these two bioconjugation approaches, making them valuable tools for various scientific and biomedical endeavours.^101,102^

**Table 2:** A comparative analysis of different properties of metal-catalyzed and metal-free bio-orthogonal click reactions

Property	Metal-catalyzed bio-orthogonal click methodology	Metal-free bio-orthogonal click methodology	Ref.
**Physical parameters**			
Energy required	They are thermodynamically driven reactions and require energy of more than 20 kcal mol^−1^, provided that metal is not used as a catalyst	Very little amount of external energy is required which can be provided by the human cells	107 and 108
Reaction time	Reaction completes within a few hours, giving the desired products	Bio-orthogonal reactions are designed to be rapid	59
Solvent used	The reaction can proceed in a variety of solvents such as organic solvents including DMF, THF, aqueous solvents, buffer systems, *etc.*	Acetonitrile or its mixture with phosphate-buffered saline was used by Bertozzi *et al.* However, recent reports have also demonstrated the completion of the reaction in human plasma, thereby further testifying the bio-orthogonality of this approach	88 and 109
Kinetics	Metal-catalyzed click reactions are much faster when compared with the metal-free bio-orthogonal click reactions	Metal-free bio-orthogonal click reactions exhibit varied kinetics depending on the methodology, *e.g.*, IEDDA possess rapid reaction rate, whereas SPAAC has a slow reaction rate, although structure-modified cyclooctynes such aliphatic and (di)benzoannulated cyclooctynes can accelerate the process	110 and 111
Order of reaction	Second	Second	112

**Chemical parameters**			
Catalyst used	Metals such as copper, ruthenium, *etc.* enhance the rate of reaction	As the reactions occur under physiological conditions, the use of metal catalysts is omitted and self-catalyzing parameters like ring strain act as the catalyst in these reactions	113 and 114

### 4.1. Mechanism

#### 4.1.1 Metal-catalyzed reactions

These reactions often involve coordination of a metal catalyst to one or more reactants, enabling activation of a specific functional group. For instance, copper-catalyzed azide–alkyne cycloaddition (CuAAC) facilitates the formation of triazole linkages by activating alkynes and azides. The metal plays a crucial role in facilitating the reaction, making it highly efficient.^103^

#### 4.1.2 Metal-free reactions

In contrast, metal-free reactions typically rely on strained rings, such as the strain-promoted azide–alkyne cycloaddition (SPAAC) or inverse electron-demand Diels–Alder reactions. These reactions do not require a metal catalyst and proceed through the release of ring strain energy, which can be a slower process.^104^

### 4.2. Selectivity and bio-orthogonality

#### 4.2.1 Metal-catalyzed reactions

While metal-catalyzed reactions can be highly selective and bio-orthogonal under optimized conditions, they are not entirely free from potential cross-reactivity in complex biological environments. Metal ions can interact with endogenous biomolecules, potentially causing interference and cytotoxicity.^99^

#### 4.2.2 Metal-free reactions

Metal-free reactions, though generally less efficient, often provide superior bio-orthogonality due to the absence of metal catalysts. They are less likely to interfere with biological processes, making them preferable for *in vivo* applications.^105^

### 4.3. Biocompatibility

#### 4.3.1 Metal-catalyzed reactions

The presence of metal ions can raise biocompatibility concerns, particularly in live cells or organisms. Strategies to mitigate this include the use of copper chelators or developing bio-orthogonal reactions with less cytotoxic metal catalysts.^99^

#### 4.3.2 Metal-free reactions

These reactions are generally more biocompatible due to the absence of metal catalysts. However, they may require longer reaction times or higher reactant concentrations to achieve the desired level of conjugation.^41^

The contrast between metal-catalyzed and metal-free bio-orthogonal click reactions underscore a fundamental choice in bioconjugation chemistry. While the metal-catalyzed reactions demonstrate notable reactivity and efficiency, their pursuit of true bio-orthogonality is hindered by the inherent challenges associated with metal-induced cytotoxicity and undesired biomolecular interactions. The need for meticulous optimization and the development of specialized ligands reflects an ongoing struggle to reconcile the benefits of metal-catalyzed reactions with the imperative for bio-orthogonality. Conversely, the metal-free click reactions exhibit superior bio-orthogonality, offering compatibility with biological systems as the absence of metal catalysts mitigates the risk of cytotoxicity, and the fine-tuning capabilities of these reactions facilitate high selectivity. Both approaches have their merits, and ongoing research continues to refine and expand the toolbox of bioconjugation chemistry.^99–102,106^

## 5. Applications of bio-orthogonal click chemistry

### 5.1. Biosensing

**Fig. 11:**
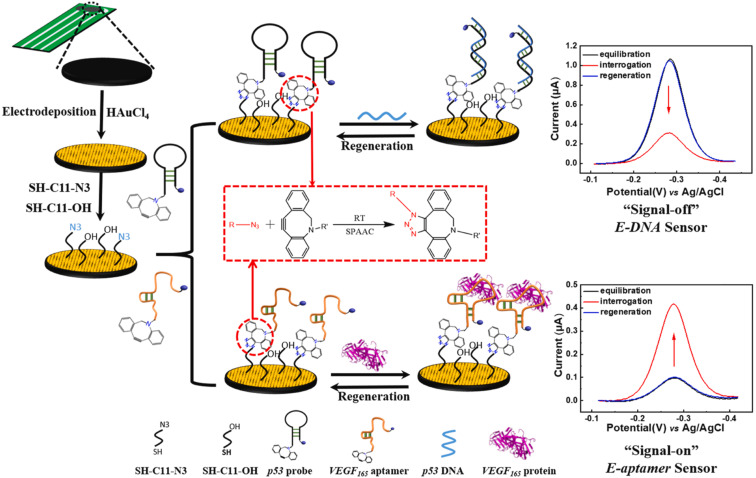
An illustration of producing biosensors *via* SPAAC to detect DNA and protein in the physiological environment. Reproduced from ref. 115 with permission from Elsevier, copyright 2022.

Fan *et al.* reported the development of electrochemical biosensors employing DNA or aptamer molecules (E-DNA/aptamer) based on target-induced alterations in the conformation of surface-immobilized oligonucleotides. A screen-printed carbon electrode (SPCE) plated with gold was utilized, which was further modified by attaching a terminal azide group by gold thiol co-adsorption technique. Dibenzocyclooctyne (DBCO) modified oligonucleotide was made to produce a 1,2,3-triazole ring through SPAAC which consequently changed the conformation of the labelled oligonucleotide on the fabricated electrode affecting the current generated through electron transfer at the electrode interface producing signals which are further analyzed. For detection of p53 DNA and VEGF165 protein, DBCO p53-MB and DBCO-aptamer-MB oligonucleotide probes were implemented as sensors on the Au-SPCE respectively, producing E-DNA/Aptamer sensors.^115^ A schematic illustration is shown in Fig. 11.

A comparable work was done by Xue Li and his team, who developed a biosensor for detecting miRNA(micro-RNA) having a basic principle of click chemistry – TdT (ccTdT) nucleic acid isothermal amplification technology wherein they used a fluorescent dye for diagnosing microRNA which is associated with a range of diseases, thereby identifying disorder in the physiological conditions. A fluorescent signal was produced due to the cleavage of the reporter gene which further assisted in accomplishing ultra-sensitive detection of miRNA.^116^

**Fig. 12:**
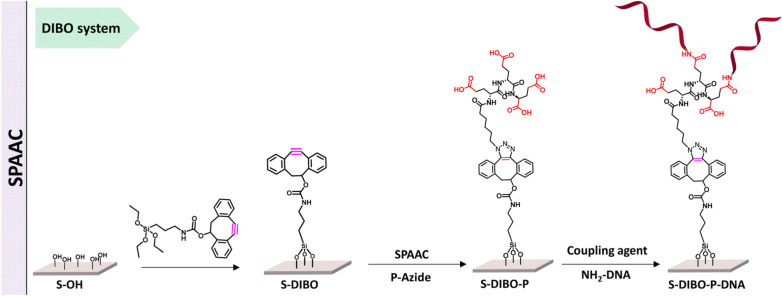
SPAAC is used to conjugate peptide with silanized *S*-alkyne. Reproduced from ref. 117 with permission from Royal Society of Chemistry, copyright 2023.

Kavand and co-workers worked on surface-based DNA-biosensor for detecting virus aiding the needs during COVID-19. They demonstrated the viability of a peptide-based branched spacer acting as an anchor to restrict DNA at a particular spot by using copper-free and copper-catalysed click chemistry as shown in Fig. 12. High DNA hybridization density was achieved by conjugating the peptides to silanized *S*-alkyne surfaces using CuAAC and SPAAC.^117^

**Fig. 13:**
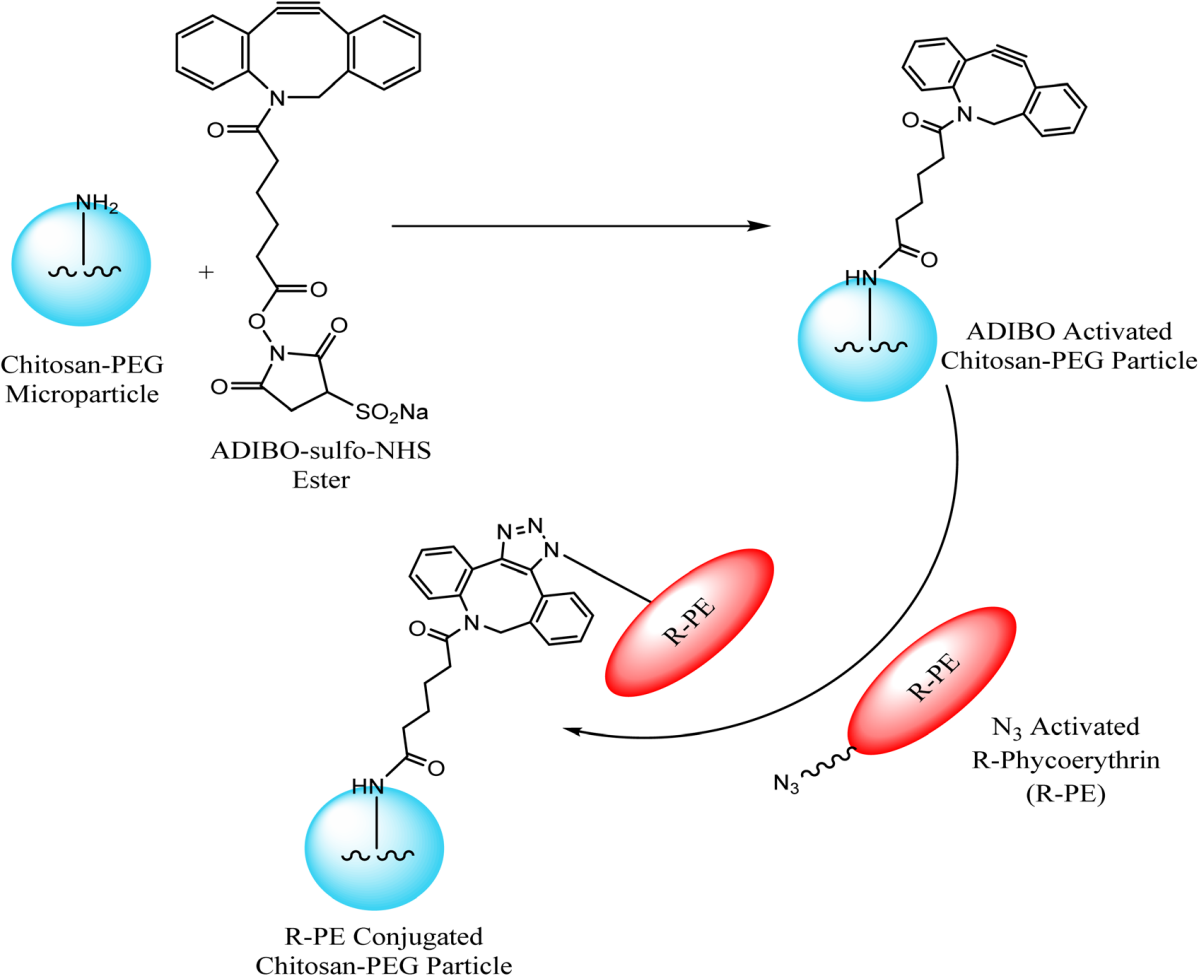
Conjugation of microparticles with *R*-phycoerythrin through SPAAC. Reproduced from ref. 118 with permission from American Chemical Society, copyright 2013.

Jung *et al.* exploited chitosan-poly(ethylene glycol) hybrid compounds, containing cyclo-octyne, for constructing highly consistent and reactive micro-particles which were subsequently conjugated with *R*-phycoerythrin – a red fluorescent protein, modified with azide group through SPAAC, thereby conjugating tunable proteins with microparticles in ambient conditions as shown in Fig. 13. This conjugation resulted from only selective proteins present in the vicinity of the particle surface.^118^

Movilli *et al.* reported that increasing the density of sterile probes over a surface area imparts enhanced sensitivity of electrochemical biosensors. They reported the design of micropillar-structured electrode, coated with gold, conjugated with poly-l-lysine (PLL). By reducing the gap between the micropillar electrodes, the density of the sensing area was increased. The PLL has branching of oligo(ethylene glycol) (OEG) and dibenzocyclooctyne (DBCO) attached, DBCO and OEG binds with azido-modified peptide nucleic acid through click reaction enhancing probe immobilizing and antifouling respectively.^119^

**Fig. 14:**
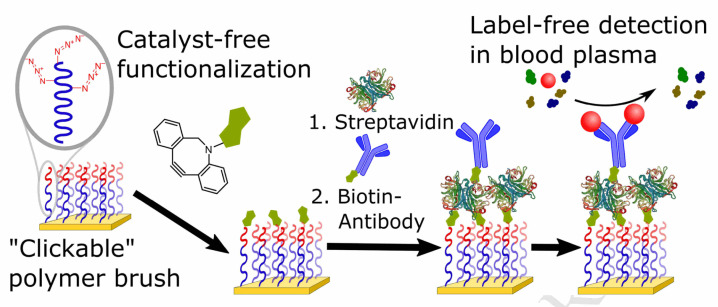
Conjugation of azide containing polymer brush with biotin using SPAAC. Reproduced from ref. 120 with permission from Elsevier, copyright 2017.

Parrillo and co-workers employed SPAAC to biofunctionalize antifouling polymer brushes containing azide groups with biotin (Fig. 14), which were able to entrap streptavidin and biotinylated antibodies. Only the end groups of the polymer brush or the side chains running the length of the top polymer block were functionalized. Surface plasmon resonance was used to immobilize concentrations of bioreceptors (streptavidin followed by biotin-conjugated proteins) and the fouling resistance from blood plasma on surface.^120^

The *O*-GlcNAcylated protein, a nutrient sensor, plays a significant part in the regulation of cellular feedback to different situations, protein turnover, DNA transcription and many others and is also a mediator in various signalling routes. However, there was a complication in the site mapping of the *O*-GlcNAc peptide, providing site-specific functional characterization, which Junfeng Ma and his team resolved through integrating chemo-enzymatic labelling, copper-free click chemistry and reductive cleavage subjecting the tagged peptide releasing from neutravidin beads to ETD-MS analysis which consequently provides a favourable tool for site-specific characterization.^121^

Bio-orthogonal reactions are also used in enhancing ultrasound molecular imaging as they provide a sturdy irreversible bond at a low cost of production. Goncin *et al.*, synthesized a targeted microbubble for P-selectin, a vascular inflammatory marker, through SPAAC to identify bowel inflammation in *vivo*. The microbubbles are injectable gas-filled bubbles that enhance the visibility during ultrasound molecular imaging *via* adhering to the disease-associated vascular biomarkers, measurements of the physiological process can be readily obtained. Previously biotin–streptavidin labelled microbubbles were employed but the latter are not compatible due to immunogenicity to human cells. However, SPAAC directly labels antibodies on the surface on microbubbles which produce similar ultrasound molecular signals as that of the biotin–streptavidin labelled microbubble, but is more physiologically suitable.^122^ Similar work was done by Slagle and his team where they primed biomarkers, αVβ3 integrin (cRGD) and VEGFR2 (A7R) proteins, cloaked microbubbles through SPAAC for diagnosing tumor angiogenesis and enhancing ultrasound molecular imaging.^123^

### 5.2. Drug delivery

Drug delivery is the process of introducing compounds of pharmaceutical relevance, such as medications and therapeutic agents, into the body in order to achieve the intended therapeutic effect. The purpose of medication delivery is to get these compounds to their intended places in the body in a regulated and efficient way. Bio-orthogonal ‘click’ chemistry plays a significant part in this process by enabling selected and regulated reactions inside biological systems, thereby facilitating the development of tailor-made delivery systems that react to particular stimuli, resulting in accurate medication release at the target site while reducing off-target effects and adds to better pharmacokinetics, controlled release, and drug solubility. Click Activated Protodrugs Against Cancer (CAPAC), which get activated only when attached to a specific tumor site, serve as an archetypal example of click-derived drugs.^124^ These CAPAC can also be modified to activate *via* continual anti-tumor feedback in a model with two tumors, which helps to account for the fact that tumor features vary across patients.^125^ Similarly, Antibody-Drug Conjugates (ADCs) are very potent in enhancing solid tumor chemotherapy, as they exhibit affinity for receptors found on solid tumors as well as stromal proteins, resulting in drug release in the click response. Additionally, the introduced ADCs have trace retention for non-target cells making them site exclusive only.^126^

**Fig. 15:**
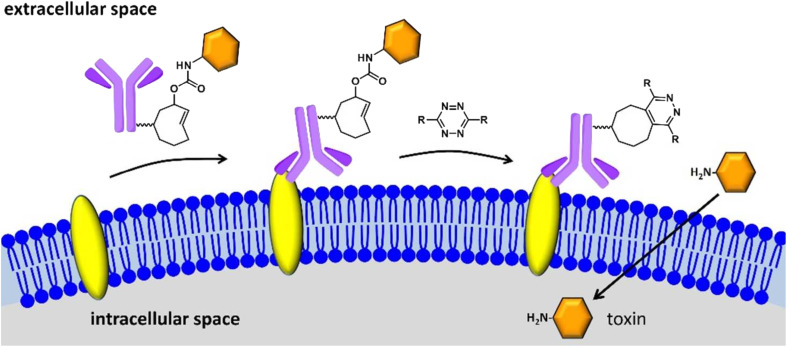
Click to release activation of antibody-drug conjugates in cancer cells. Reproduced from ref. 128 with permission from American Chemical Society, copyright 2016.

Researchers have also initiated the use of IEDDA pyridazine elimination reaction, a type of click reaction with high kinetics, for expanding the click-triggered release drug realm,^127^ as shown in the Fig. 15. Furthermore, IEDDA reactions are effective in unleashing alcohol-bearing drugs and medication *in vivo*. Such a strategy can be securely implemented in physiological environment for deprotecting amino acids, carbohydrates, fluorophores, and even drugs, activating toxic derivatives at regions where required, keeping others in the clear.^128^

Anticancer nanomedicine was synthesised by Wang *et al.* by integrating a series of reactions, out of which one was the copper-free click chemistry. A polymeric prodrug based on polyactide (norbornene functional) was synthesized, developed through heat-promoted 1,3-dipolar cycloaddition click reaction, having functionalized paclitaxel, chemotherapy medication to treat various types of cancer, conjugated covalently with water miscible polymer carrier *via* acid-sensitive hydrazine bonds, releasing paclitaxel in low pH. The conjugated structure was characterized by FTIR, ^1^H NMR and gel permeation chromatography, showing high effectiveness towards cancer cells.^129^

Massaad-Massade *et al.*, proposed an ideology to exploit polyisoprenoid chains, squalene (SQ) and solanesol (SOLA), to act as nanocarrier and to deliver siRNA counter to TMPRSS2-ERG, an oncogene found in prostate cancer, reducing oncoprotein expression. The conjugation between siRNA and the polyisoprenoid was accomplished through copper-free click chemistry; as a consequence of biocompatibility, minimal side-products and higher yield was observed.^130^

Hood and his companions crafted click radio immunoliposomes which have been validated as resourceful in targeted vascular drug-delivery agents as well as in molecular imaging. The radioactive immunoliposomes were synthesized *via* copper-free click chemistry wherein the liposomes were conjugated with monoclonal antibodies (Ab) or their single chain variable fragments (scFv) to target specific platelet-endothelial cell adhesion molecule (PECAM-1) and intracellular adhesion molecule (ICAM-1) where their biodistribution was analyzed using isotope detection and non-invasive imaging in the organ.^131^

Amphiphilic chitosan derivatives are chitosan molecules that have been modified with both hydrophilic and hydrophobic groups. This gives them the unique ability to interact with both aqueous and non-aqueous environments, making them useful for a wide range of applications and have recently gained attention as a drug carrier. Tao *et al.* took advantage of copper-free click chemistry for preparing these chitosan derivatives taking into account several advantages of the technique such as high productivity and eco-friendly characteristic.^132^

**Fig. 16:**
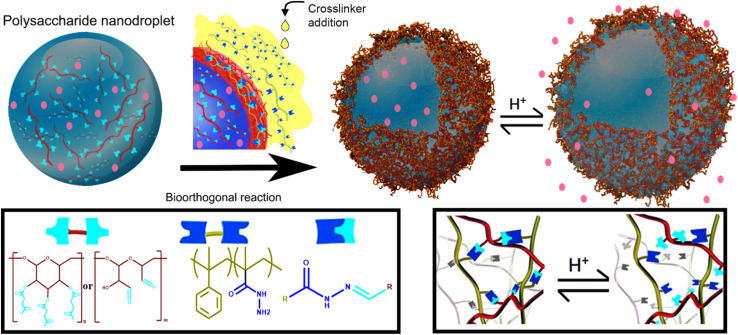
Synthesis of nano-capsule using bio-orthogonal click chemistry. Reproduced from ref. 133 with permission from American Chemical Society, copyright 2020.

Nanocapsules working as a delivery system were prepared by Alkanawati and her co-worker utilizing bio-orthogonal click chemistry wherein a network of hydrazone was produced on the interface of aqueous nanodroplets, polysaccharide in miniemulsion, reacting with functionalized polyhydrazine resulting in formation of pH-responsive nanocapsules releasing the encapsulated payload in different pH environments with negligible amount of cytotoxicity.^133^ Illustration of the process is shown in Fig. 16.

Chiu and his companions rendered large-pore mesoporous silica nanoparticles which were integrated with different organic functional groups through a co-condensation strategy which was eventually subjected to copper-free click chemistry by incorporating DBCO into the system to craft a cargo-delivery system.^134^

The Natural Killer (NK) cells are known for their ability to recognize and eliminate virus-infected cells and cancer cells without prior exposure or specific recognition of antigens. However, their ability is concealed over time making them ineffective for therapeutic use. Deng and his team worked to craft pH-responsive self-clustering nanoparticles which could co-deliver chemotherapeutic doxorubicin (DOX) with NK cells-based immune-chemotherapy *via* copper-free click chemistry wherein they utilized polycaprolactone-poly(ethylene glycol) micelles conjugated with DBCO or azido (N_3_) and coated with acid-cleavable PEG5000. They remained unaltered in neutral physiological circulation while triggering copper-free click reaction when subjected to acidic conditions of tumor cells aggregating the nanoparticles with increased retention capabilities.^135^

Pramanik *et al.* worked with cubosomes derived from space group *Im*3*m* for utilizing them as a targeted drug delivery vehicle, and for doing the same they functionalized the surface with Affimer protein, engineered with DBCO, *via* copper-free click chemistry, thereby increasing the specificity of the system towards the antigens of colorectal cancer cells, both *in vivo* and *in vitro*, and delivering copper acetylacetonate, anticancer drug.^136^

Transfection is a biological technique used to introduce foreign genetic material, such as DNA or RNA, into eukaryotic cells. The process of transfection allows researchers to study the effects of specific genes or gene products in a controlled environment. It is widely used in molecular biology and genetic research to investigate gene function, gene regulation, and protein expression. O'Brien and his team used bio-orthogonal click chemistry which they called SnapFect and also used integrated liposome fusion and cell engineering to transfect nucleic acid to targeted cell population. They modified the surface of the targeted cell population with active bio-orthogonal molecule and induced an interfacial click reaction with the complementary bio-orthogonal active nucleic acid delivering it to the specific cell population. This technique has widespread utility and can be performed in different cell culture media on multiple types of cells.^137^

**Fig. 17:**
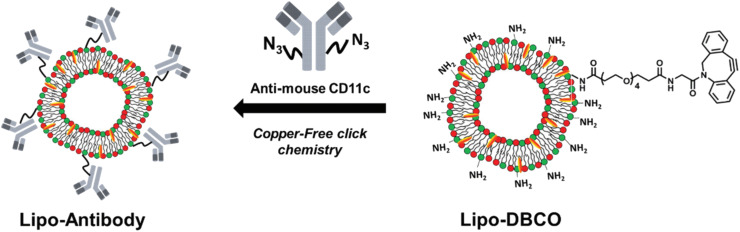
Antibodies delivery on the surface of liposomes using SPAAC conjugation. Reproduced from ref. 138 with permission from the Royal Society of Chemistry, copyright 2019.

Site-specific conjugation is a key characteristic of bio-orthogonal reactions that have been exploited for various binding reactions. Gai *et al.* utilized the reaction for conjugating antibodies at specific sites. They modified the antibodies with the azide segment and linked them with amino groups having strained cyclooctyne over the surface of liposomes *via* SPAAC resulting in the formation of 1,2,3-triazole ring, as shown in Fig. 17, and delivering the antibodies at a specific site.^138^

For studying pharmacokinetics and controlled drug release, Ediriweera and co-workers implemented ‘click-to-release’ strategy and developed a pro-drug triggered on antibody-drug conjugation for cancer treatment. Pre-targeted bio-orthogonal drug and quantitative imaging together had shown efficiency in delivering precise amounts of drug at targeted sites in animals, provided technique limited to nanomedicines. Hyperbranched polymer system for increasing effectiveness of the drug delivery system.^139^

### 5.3. Labelling

Zhang and his companions reported bio-orthogonal mechanisms complexes for dragging out targeted microRNA genes in a live cell through artificially introducing DBCO at 3′ end of microRNA attracting Ago2 protein consequently forming RNA-induced silencing tagging microRNA gene simplifying the complicated chore.^140^

**Fig. 18:**
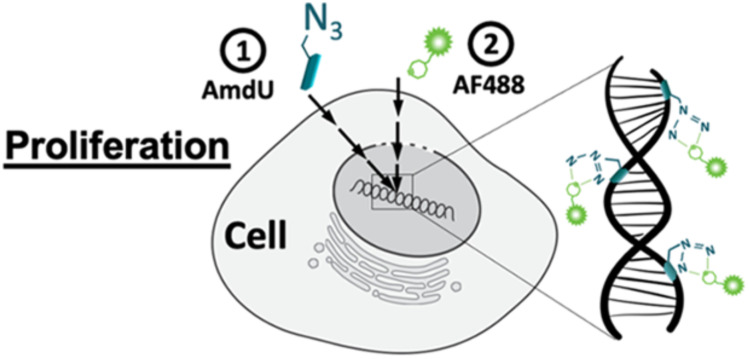
Labelling chondrocytes using SPAAC with fluorophore. Reproduced from ref. 141 with permission from American Chemical Society, copyright 2022.

Acute inflammation occurs after joint injuries causing degeneration of chondrocytes leading to lowering extracellular matrix levels and also reduces the collagen and glycosaminoglycan content triggering mechanical damage in the cartilage. Porter *et al.*, utilized bio-orthogonal click chemistry for quantifying the proliferation rate of the chondrocytes and analyzing the destructive effects. For labelling the cells azide-tailored nucleoside, 5-(azidomethyl)-2′-deoxyuridine (AmdU), was added in the culture medium which was taken up by the cells and incorporated with the DNA or recently synthesized protein molecule which later on coupled with the DBCO-modified fluorophore, thereby labelling the cells (Fig. 18).^141^

**Fig. 19:**
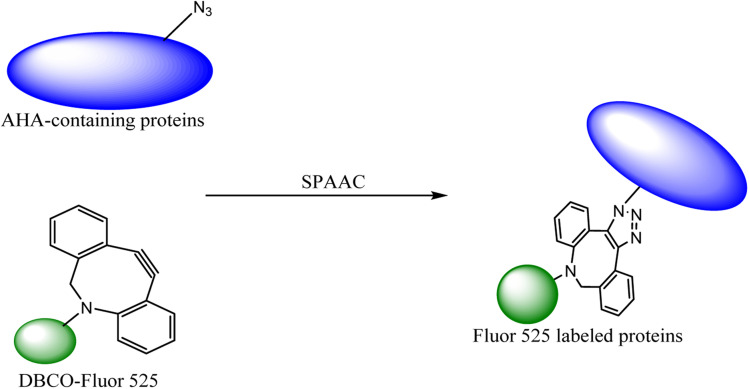
Azidohomoalanine (AHA) containing protein was labelled through metal-free click chemistry.^143^ Reproduced from ref. 143 with permission from American Chemical Society, copyright 2017.

The labelling of the physiological molecules is a vital technique for studying their dynamics and correspondingly taking action for desired consequences. This can be achieved by using bio-orthogonal reactions, one such example was previously carried out by Macias-Contreras and his team where SNAP/CLIP tag technology was incorporated with bio-orthogonal reaction labelling simultaneously two proteins of concern within active cells. The SNAP and CLIP tags were genetically incorporated *via* azide–alkyne cycloaddition at the subcellular site. The successive bio-orthogonal reactions with fluorophores lugging functionalities increased the efficiency and potential of SNAP/CLIP tags to garnish the biological cells, utilizing the technique for radioactive tracing, spin labelling, and many more.^142^ Similar work was done by Huang and his co-worker wherein they approached to dually labelled viruses, obligate parasites, by merging protein and DNA metabolic incorporation with two bio-orthogonal reactions in a single cell as shown in Fig. 19.^143^

**Fig. 20:**
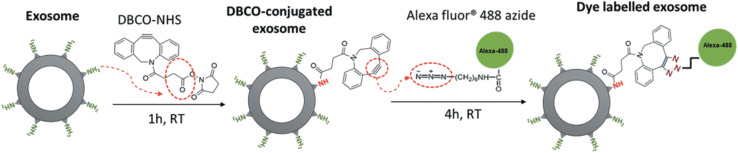
Fluorescent labelling of exosomes through bio-orthogonal click chemistry. Reproduced from ref. 144 with permission from Taylor & Francis, copyright 2020.

Similarly, Xu and co-workers employed the same bio-orthogonal click reaction to fluorescently label exosomes, used in therapy for pancreatic cancer, without any alteration in the parent cell as shown in Fig. 20. Labelling of exosomes enabled to trace their uptake by pancreatic carcinomas and therefore aided to evaluate the dose and incubation time of exosomes.^144^

**Fig. 21:**
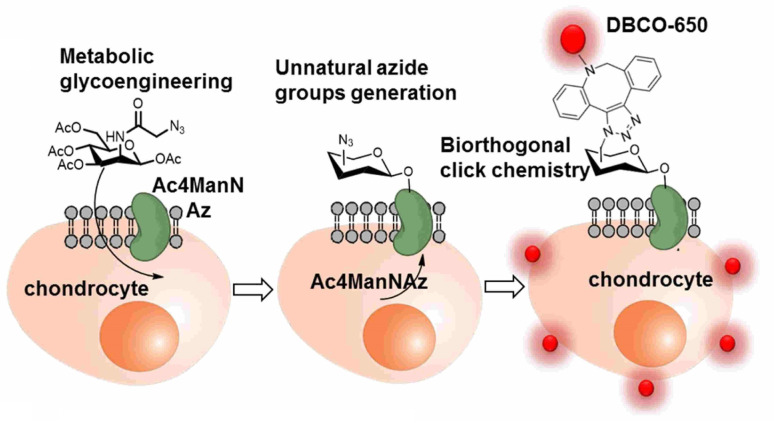
An illustration of bio-orthogonal conjugation between synthetic azide group of chondrocytes and DBCO-650 labelling the cell. Reproduced from ref. 145 with permission from American Chemical Society, copyright 2016.

The labelling and tracking of the cells are also vital in the cell therapy treatment of chronic degenerative diseases, such as arthritis, neurodegeneration, cardiac diseases and many others, involving stem cells and meiotic cells. One such efficient labelling and tracking technique was proposed by Yoon and his companions where they first introduced a synthetic azide group to the chondrocytes through metabolic glycoengineering by providing Ac_4_ManNAz in the cell culture medium and subsequently conjugating it with near-infrared fluorescent (NIRF) dye-tagged dibenzyl cyclooctyne (DBCO-650) *via* bio-orthogonal copper free click chemistry, as shown in Fig. 21, where the conjugated cells were tracked by NIRF imaging system.^145^

Another site-specific radioactive labelling was accomplished by Jeppesen and his co-workers where they conjugated azide group which was artificially incorporated into the targeted molecule; incorporation was effortless and unreactive toward other biomolecules, with the NOTA moiety (a cyclic polydentate bifunctional chelator with robust kinetic stability) through SPAAC, thereby producing a conjugate which was later labelled with ^64^Cu. The site-specific radio-labelled conjugate produced was steady and unreactive towards other physiological molecules making the labelling method effective and reliable and was further examined *via* Positron Emission Tomography (PET).^47^

Similarly, site specific labelling of antibodies through bio-orthogonal click chemistry was proposed by Wu and his team to acquire the perks of biochemical qualities and therapeutic indices of the antibody conjugates.^146^

**Fig. 22:**
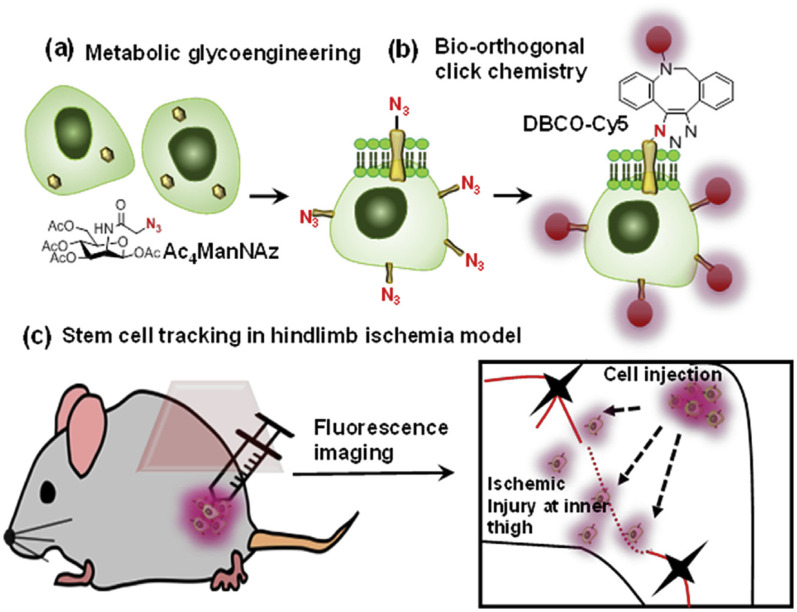
An illustration of the strategy for tracking stem cells through copper-free click chemistry. (a) Incorporation of azide group through metabolic glycoengineering, (b) fluorescent labelling of modified Ac_4_ManNAz *via* biorthogonal click chemistry and (c) injecting triazole derivative to distinguish transplanted stem cells. Reproduced from ref. 147 with permission from Elsevier, copyright 2016.

Bio-orthogonal copper-free click chemistry was also utilized by Lee and companions to distinguish transplanted stem cells from host cells during stem cell therapy. They treated adipose-derived mesenchymal stem cells with tetra-acetylated *N*-azidoacetyl-d-mannosamine (Ac4ManNAz) and incorporated artificial azide and later conjugated them with dibenzylcyclooctyne-conjugated Cy5 (DBCO-Cy5), labelling them with fluorescently as shown in Fig. 22. These labelled compounds, introduced in the hindlimb ischemia mice model, were traced and analyzed through an optical imaging system to appreciate therapeutic effect.^147^

**Fig. 23:**
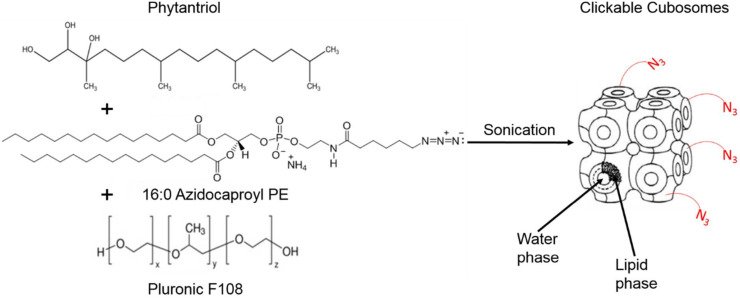
Cubosome functionalised utilising metal-free click chemistry. Reproduced from ref. 148 with permission from American Chemical Society, copyright 2018.

Cubosomes are nanostructured particles composed of lipid bilayers arranged in a three-dimensional cubic lattice. Alcaraz *et al.*, functionalized cubosomes with azide or DBCO (as shown in Fig. 23) and examined their click reactivity with the complementary part using cryo-TEM, small angle X-ray scattering and thriving light scattering characteristics. Furthermore, their clickability was analyzed using a counter dye which exhibited an insignificant change in structure, size, and shape demonstrating the stability of cubosomes in physiological conditions and proficiency to accomplish click reaction delivering drug and metabolic labelling.^148^

Vabbilisetty *et al.* also used bio-orthogonal click chemistry for labelling cells with lipids of choice, helping them in examining and re-engineering the cell's surface for appropriate utilization. The site-specific characteristic of the bio-orthogonal click chemistry enables them to conjugate lipids such as phosphatidylethanolamine–poly (ethylene glycol)–DBCO and cholesterol–PEG–DBCO at the targeted cell surface. Confocal microscopy and flow cytometry analysis confirmed the conjugation of the reactive lipid species making the technique ideal for incorporating biomolecules over different cell surfaces.^149^

G-quadruplex is a unique secondary structure that can form in nucleic acids, particularly in DNA and RNA molecules. It is characterized by a specific arrangement of guanine (G) bases in a tetrad, which forms a stable, stacked structure. In addition to this, they can be linked with small reporter molecules and can be further used in tracking both *in vivo* and *in vitro*. Lefebvre *et al.* investigated the cellular localization of PhenDC3 by taking advantage of click chemistry. They developed PhenDC3 clickable derivatives of alkyne and azide and implemented them for tracking. It was demonstrated that labelling the cells using copper-free SPAAC was more reliable than the one performed *via* CuAAC.^150^

For the detection of carcinoembryonic antigen (CEA), Xiang research group evolved a technique wherein graphene oxide functionalized with azide (GO-N_3_) were made to conjugate with their counter-part, *i.e.*, DNA labelled through carbon dots (CDs) *via* copper-free click chemistry. The reported conjugation extinguished the fluorescence of carbon dots as a consequence of energy transfer due to fluorescence resonance. The addition of carcinoembryonic antigen restored this fluorescence, enabling their tracing.^151^

**Fig. 24:**
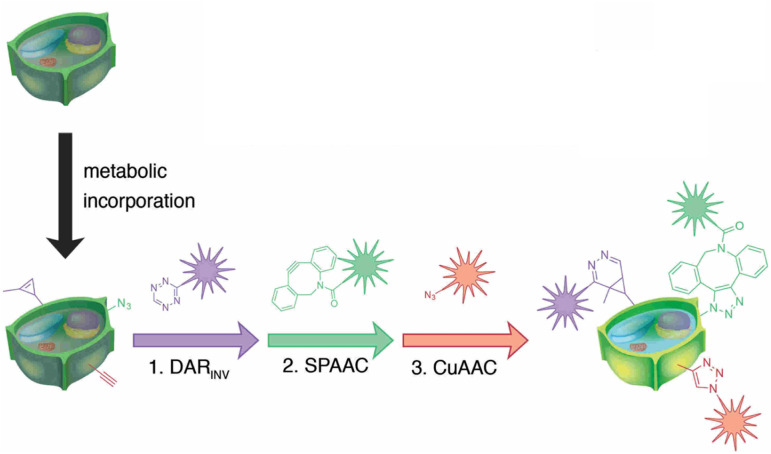
Combination of CuAAC, SPAAC and DAR_INV_ for labelling three different lignin monomeric units. Reproduced from ref. 152 with permission from John Wiley and Sons, copyright 2018.

Simon and co-workers also utilized click chemistry for *in vivo* triple labelling approach in plant tissues to detect *de novo* synthesis of lignin. They used a combination of CuAAC, SPAAC and Diels–Alder reaction (DAR_INV_) to track down the integration of three different monomeric units of lignin into the cell wall (Fig. 24). The technique is an ideal imaging toolbox for detecting three different mimics all at once.^152^

Haun *et al.* targeted *N*-linked glycoprotein present over the carcinomas of the pancreas to diagnose and treat pancreatic cancer. They labelled the glycoprotein with biotin *via* bio-orthogonal click chemistry and then analyzed them using mass spectroscopy which revealed some newly reported proteins, thereby demonstrating that glycoprotein profiling can be utilized for cell protein surface analysis.^153^

Labelling and detection of biomolecules serve several significant purposes, each contributing to our understanding of biological processes and the development of various applications. Kang and companions attempted to enhance the classic bio-orthogonal labelling technique by using multi-fluorinated aryl azides and developing reactive H_2_S fluorescence probes. Substitution of fluorine atoms in aryl azides, enhanced the kinetics of H_2_S facilitated reduction, owing to the fact of small size and high electro-negativity of fluorine atoms, thereby improving the rate of labelling process. This advanced SPAAC conjugation technique is also applicable in transient molecules taking-in detection of radio-isotopes.^154^

Li and group developed a two-fold strategy involving metabolic engineering and metal-free click chemistry for the detection, segregation and elimination of toxic bacteria present in mammalian cells. They tagged the cell wall of bacteria with reporter molecules containing the alkyne group through metabolic engineering. The artificial group engineered over the surface of bacteria had the potential to conjugate with nanoparticles and alter with the azide group through bio-orthogonal click chemistry. The adduct produced could be segregated from other living mammalian cells and could further be eliminated using some specific drugs *in vivo* treating bacterial infection.^155^

A similar attempt was made by Kim and companions wherein they developed a strategy for imaging the quantity of drug binding with targeted molecules inside cytoplasm using spatially localized protein expression in combination with bio-orthogonal labelling method. They worked on dasatinib, a multi-target drug and kinase inhibitor, and altered it with *trans*-cyclooctyne and coupled it with the targeted molecule present in the cytoplasmic compartment through SPAAC. Bio-orthogonal labelling followed by imaging of the dasatinib molecule gave kinetics of the binding reaction.^156^

RNA serves as an intermediary between DNA and protein synthesis and it is therefore essential to interpret the structure and function in different physiological conditions giving rise to necessary labelling. George and team used bio-orthogonal click chemistry, chemo-selective characteristics, for labelling RNA. The technique can also be utilized on other biomolecules.^157^

Metabolic oligosaccharide engineering (MOE) is used in studying polysaccharides in eukaryotes as well as in bacterial cell walls, however, the technique fails to detect virulent bacterial capsules and K1 polysialic acid which provide resistance to bacteria from the immune system. Aiding this, Rigolot research group fabricated a fluorescence microplate assay, detecting K1 capsule and its biosynthetic pathway, using a combination of MOE and bio-orthogonal click chemistry. *N*-Acetylmannosamine (ManNAc), precursor of K1 capsule, was armed with alkyne and azide counterpart and incorporated into bacteria and later conjugated with fluorophore labelling the bacterial capsule through bio-orthogonal click chemistry and enhancing the tracing of bacteria.^158^

**Fig. 25:**
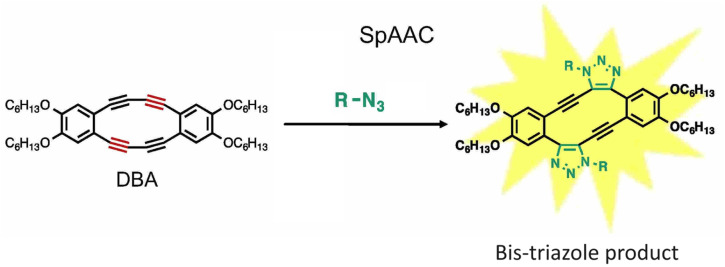
Bis-triazole product is formed when octadehydrodibenzo [12] annulenes undergo strain-promoted-azide–alkyne cycloaddition. Reproduced from ref. 159 with permission from John Wiley and Sons, copyright 2019.

Fukushima *et al.* reported strain promoted double azide addition reaction producing two triazole rings in the same molecule. They made use of octadehydrodibenzo [12] annulenes (DBAs), derivative of 1,2-diethynylbenzene, and reacted it with organic azides producing bis-triazole (Fig. 25). Further, X-ray crystallography and ^1^H NMR confirmed the formation of the product. This bis-triazole product emitted green fluorescence when irradiated with UV light and therefore could be used in the labelling of azidated molecules, and hence the reaction has the potential to emerge as an important toolbox in material science.^159^

Liao and team developed a strategy derived from liver sinusoidal endothelial cells (LSEC) to enhance liver engraftment proficiency and tagged them with a fluorescent probe, indocyanine green – active in the near-infrared range *i.e.*, from 12500 cm^−1^ to 4000 cm^−1^. LSCE-altered peptides and the fluorescent dye were implanted over adipose tissue-derived mesenchymal stem cells *via* bio-orthogonal click chemistry and metabolic glycoengineering for *in vivo* tracing and binding boosting the cell therapy.^160^

Necrosis avid agents (NACAs) are a group of imaging compounds that bind specifically to necrotic tissue and are used in imaging of various diseases such as cancer, stroke, infection, and many more. Generally, they are used in conjunction with magnetic resonance imaging (MRI) and single-photon emission computed tomography (SPECT). Jiang and companions developed a bio-orthogonal click chemistry-based elementary probe by altering rhein with *trans*-cyclooctene, showing affinity with necrosis genetic material *i.e.*, DNA and rRNA.^161^

### 5.4. Hydrogels

**Fig. 26:**
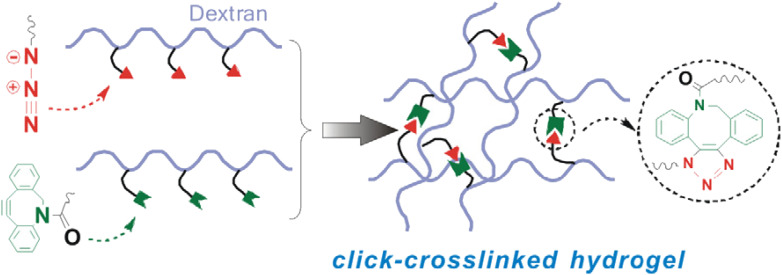
An illustration of crosslinking between Dex-N_3_ and Dex-ADIBO *via* SPAAC. Reproduced from ref. 162 with permission from Elsevier, copyright 2017.

Azadibenzocyclooctyne modified dextran (Dex ADIBO) was coupled with azide-modified dextran (Dex-N_3_) through strained promoted ADIBO-azide click chemistry by Wang *et al.* to prepare a hydrogel (monomeric unit shown in Fig. 26). The properties of synthesized material could be controlled by altering the concentration of the polymer and the degree of substitution of conjugated dextran. The strain-promoted cyclooctyne azide cycloaddition, which takes place in mild conditions and is inert in a physiological environment, is a practical route to produce injectable hydrogels that promote *in vivo* culturing of chondrocytes and their matrix production, thereby aiding tissue engineering.^162^

The silicone elastomers are biocompatible and user-friendly, however, are associated with the hydrophobic amino acid and lipids absorbing surface, reducing the ability of their application in ocular operation. Lasowski and his team synthesized silicone hydrogels by installing moiety possessing hydro-affinity through metal-free click chemistry wherein polydimethylsiloxane (PDMS) containing terminal alkyne crosslinked with reactive azido group of PDMS-*g*-PEG surfactants producing co-polymer, thereby increasing the efficiency of drug release. The protein fouling and sustained release kinetics wettability are characteristic features of the co-polymer improving the biomaterial.^163^

Hydrogels with covalent cross linkages degenerate despite their tunability and stability in the physiological environment, affecting the normal functioning of cells. To surmount this difficulty Zhan *et al.* manufactured poly(ethylene glycol) based dually cross-linked hydrogels containing an equal number of covalent and non-covalent linkages produced through interaction between calcium ion and phosphonate groups, with storage moduli less than 2000 Pa. These dually linked hydrogels showed 41 to 96% recovery to their original mechanical properties after two consequent degeneracy and also showed a highly biocompatible nature.^164^

Similar work was done by Arkenberg and the team where they employed bio-orthogonal click reaction, aided with enzymatic reaction and covalent adaptable chemistry, to develop biomimetic hydrogels installing specific biomimicry in extracellular fluid. Dual cross-linked hydrogels had better mechanical properties and stability, because of tuning with reversible and irreversible chemistry, allowing them to flexibly conjugate with different biomolecules in extracellular fluid.^165^

**Fig. 27:**
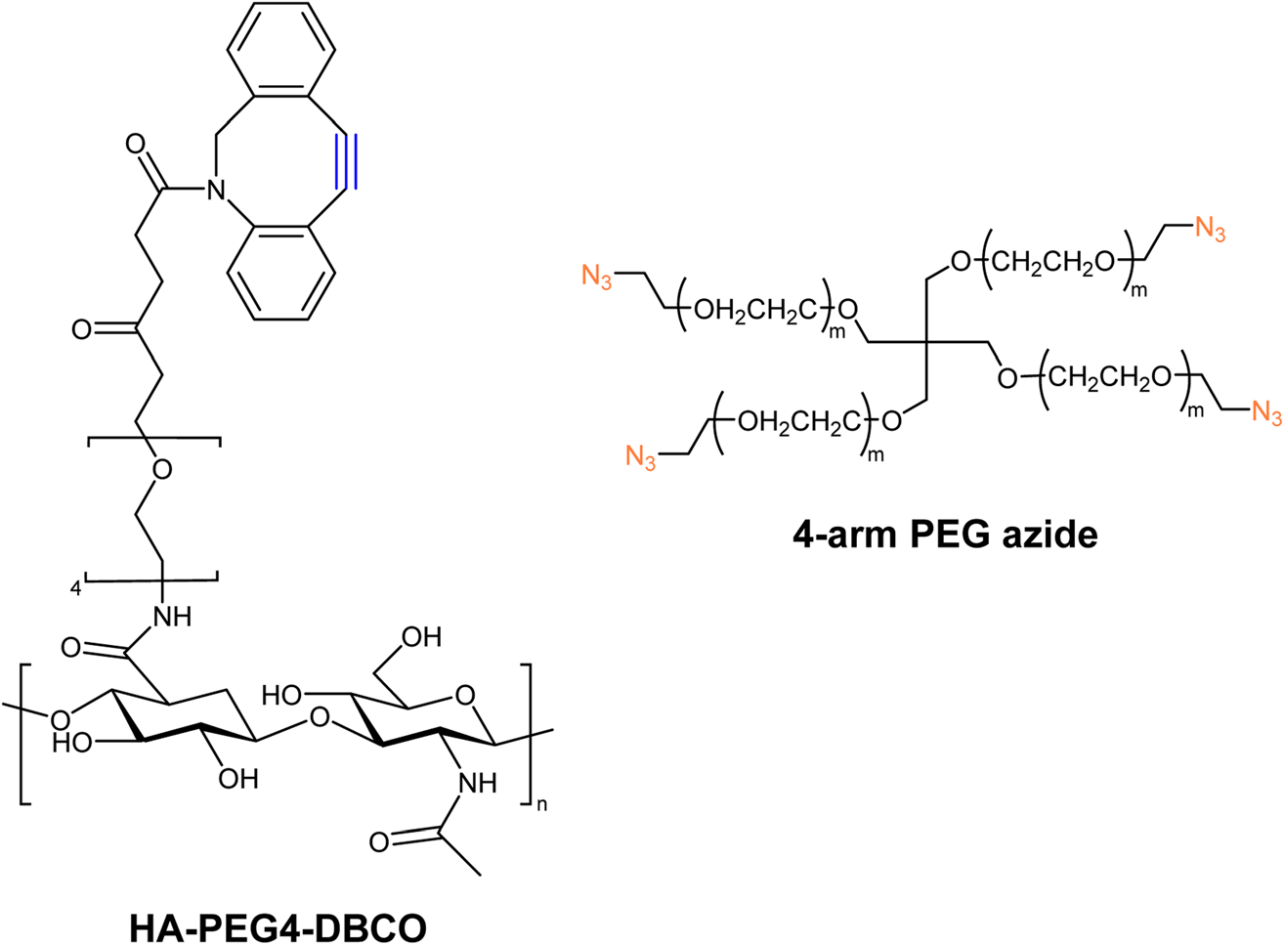
Structure of HA-PEG4-DBCO alkyne and 4-arm PEG azide utilized for the synthesis of corresponding hydrogels.^166^ Reproduced from ref. 166 with permission from Royal Society of Chemistry, copyright 2010.

Han and companions exploited bio-orthogonal click chemistry for synthesising cross-linkable hyaluronic acid derived hydrogels and utilized the hydrogel as injectable scaffolds in the engineering of cartilage tissue. They modified hyaluronic acid (HA) with DBCO, producing HA-PEG4-DBCO, and conjugated it with 4-arm PEG azide through SPAAC (Fig. 27). The concentration of counter-parts modulates the elastic modulus of the product and thereby can be altered according to requirements.^166^

### 5.5. Materials synthesis

Surface-active microparticles were synthesised by Walden *et al.*, where they functionalized a model protein with DBCO and triggered a reaction with the counter-part *i.e.*, azide-terminal polycaprolactone surface, immobilizing the fabricated microcarrier, *via* copper-free click chemistry in a physiological environment with high proficiency. This methodology can also be used to immobilise other bioactive molecules to the surface of microcarriers which can be used in cell engineering as well as in drug delivery.^167^

**Fig. 28:**
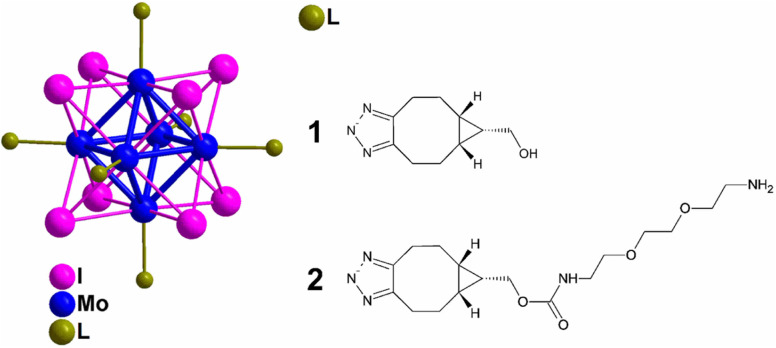
Molybdenum-iodine nanocluster stabilized by triazolate ligand 1 and 2. Reproduced from ref. 168 with permission from American Chemical Society, copyright 2022.

Kirakci *et al.*, utilized copper-free click chemistry to synthesise a singlet oxygen photosensitizing complex consisting of octahedral molybdenum-iodine nanocluster, stabilized by triazolate apical ligands, by making use of [Mo_6_I_8_(N_3_)_6_]^2−^ and bicyclo[6.1.0]nonyne (BCN)-functionalized apical ligand as shown in Fig. 28. The prepared nanocluster works by directly targeting the cell membrane wiping out the need of endocytosis to take in the drug and also cause a distinguishable blue-light phototoxic effect counter to oncocytes. Further, the nanocluster shows a dominant effect, consequent to apical ligand, when compared with negatively charged parent chain and also the nanoaggregates complex were self-disabling due to hydrolysis preventing post-treatment side effects boosting photodynamic efficiency and intensifying their application for cancer theranostics.^168^

**Fig. 29:**
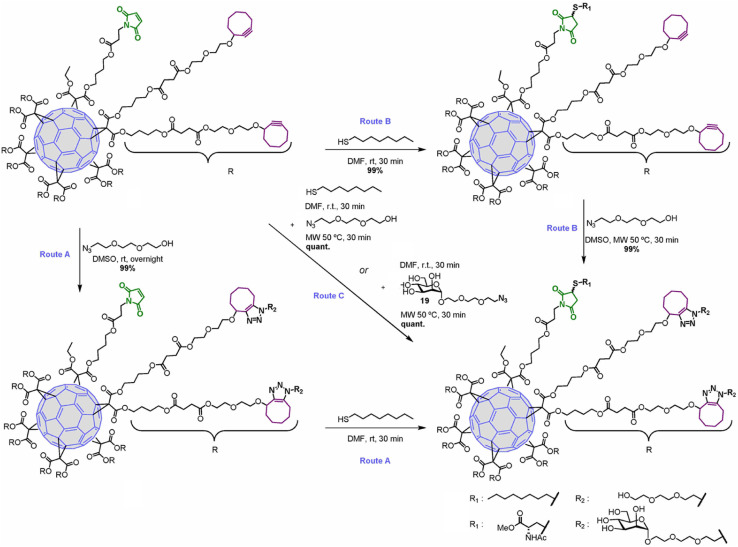
One-pot conversion of hexakis adducts of [60]fullerene into multivalent system through SPAAC in different reaction conditions. Reproduced from ref. 169 with permission from American Chemical Society, copyright 2018.

Hexakis-adducts of [60]fullerene is used in many photovoltaic devices and also have many biological implementations and a multivalent system based on hexakis-adducts of [60]fullerene was synthesised by Soriano and his group where they utilise copper-free click chemistry and follow a one-pot conversion and to carry out the same. They generated an asymmetric derivative of the hexakis-adducts of [60]fullerene with one maleimide and ten cyclooctynes to carry out the approach as shown in the Fig. 29 and created a physiologically compatible hexakis adduct in conjugation with ammino acid and saccharide.^169^

**Fig. 30:**
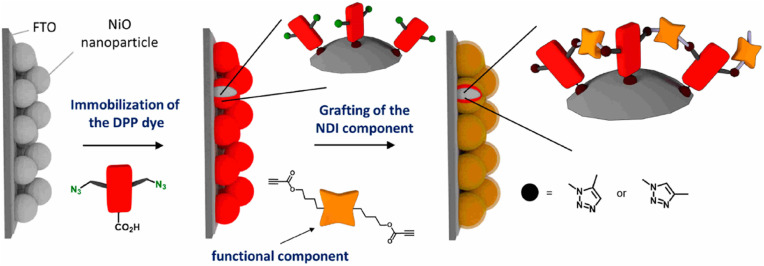
Conjugation between NPI and DPP using copper-free click chemistry. Reproduced from ref. 170 with permission from American Chemical Society, copyright 2021.

Bentounsi and his team constructed multicomponent hybrid dye-sensitized photoelectrochemical cells on electrode surface using copper-free click chemistry. The reaction between the activated alkyne, a derivative of naphthalene diimide (NDI) consisting two propiolic esters, and the azide group containing diketopyrrolopyrrole (DPP) dye, on the surface of NiO photocathode resulted in the formation of the 1,2,3-triazole ring and hence conjugating DPP dye on the surface of photocathode as illustrated in Fig. 30.^170^

**Fig. 31:**
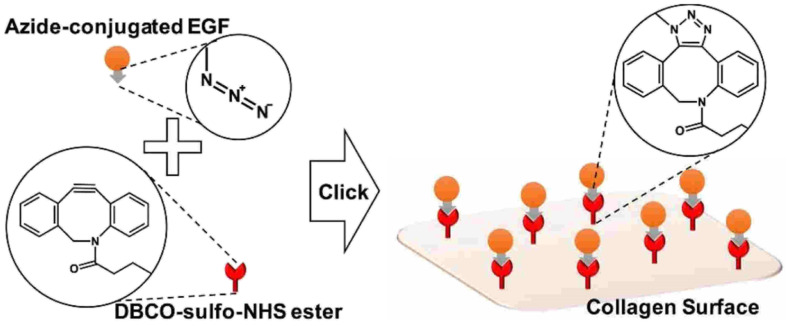
Conjugation of EGF with collagen surface *via* SPAAC enhancing the proliferation of the epithelial cells. Reproduced from ref. 171 with permission from American Chemical Society, copyright 2017.

Jong Lee and his group worked on the adhering epidermal growth factor (EGF) onto the surface of collagen, boosting proliferation and anchoring factors of the corneal epithelial cells. They proceeded by attaching the azido group over the surface of EDF and covalently coupling it with the DBCO group present on the collagen surface through SPAAC (as depicted in the Fig. 31), immobilizing the EGF which was analysed using ELISA, XPS and SPR spectroscopy and consequently producing a biocompatible system, with low cytotoxicity and enhanced proliferation rate, *in vitro*.^171^

Bio-orthogonal elastin-like polypeptide (ELP) support structure with periodic non-canonical l-azidohomoalanine amino acids in the guest residue position was constructed by Tien Ta *et al.*, using SPAAC wherein they utilized azide conjugated elastin-like polypeptide as the modified specific conjugation site with the DBCO anchored antibodies, for organizing multiprotein complexes in a definite configuration. ELP is also conjugated with the sortase A and ybbs tags providing supplementary space for derivatizing small molecules and also assisting reaction to be carried on in a one-pot approach. Multi-antibody ELP system enhances the immune-specific biosensors and multi-channel cell labelling.^172^

**Fig. 32:**
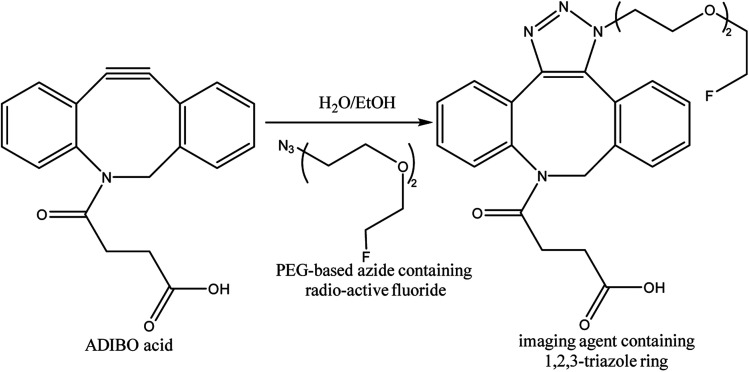
SPAAC used in synthesis of radioactive fluoride possessing imaging agent.^173^ Reproduced from ref. 173 with permission from American Chemical Society, copyright 2020.

One-bead one-compound (OBOC) library derived from peptide-based imaging agents was designed by Murrell *et al.*, using copper-free click chemistry wherein they utilized cyclooctyne-altered peptide *i.e.*, azadibenzocyclooctyne acid and coupled it with azide modified ^18^F-synthetic group, PEG-based, resulting in a radiotracer (Fig. 32) serving in PET imaging.^173^

Zhang and his team developed site-specific recombinant thrombomodulin using copper-free click chemistry wherein they adjoined azidohomoalanine at C-terminal and coupled it with DBCO containing glycopolymer resulting in end to end conjugation of thrombomodulin and glycopolymer, analysed using SDS-PAGE, western blot, and protein C activation assay, simplifying production of recombinant thrombomodulin based anticoagulate agents in physiologically favoured conditions.^174^

Copper-free click chemistry was employed by Li *et al.*, to synthesis thermo-responsive DNA copolymer where they coupled DBCO dUTP, which is an artificial nucleotide, modified DNA molecule generated using Polymerase Chain Reaction (PCR) with poly *N*-isopropylacrylamide (PNIPAM) serving as thermo-responsive side chain. Analysis using single-molecule fluorescence microscopy showed that the product is influenced by temperature, molecular weight and density of the branched chain.^175^

**Fig. 33:**
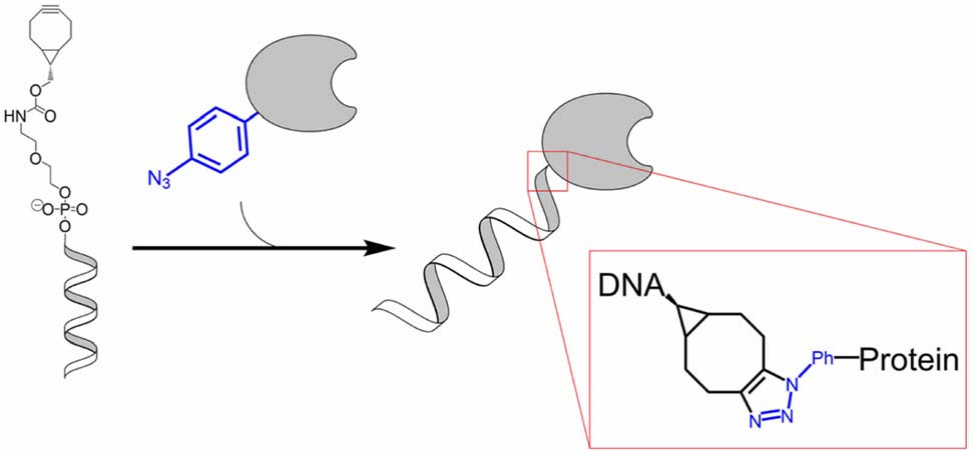
SPAAC used in conjugation of ssDNA and protein of interest. Reproduced from ref. 176 with permission from American Chemical Society, copyright 2020.

Multicomponent assembly of single-stranded (ss) DNA and protein of interest (POI), such as enzyme, was created by Marth and team by utilising SPAAC wherein they conjugated ssDNA having terminal strained bicyclononyne with *p*-azido-l-phenylalanine, genetically encoded at specific site, present on peptide chain, as shown in Fig. 33, without affecting the functioning, even if nearer to active site. The approach enhances strikingly rate of multiple enzyme system, enabling better understanding of the system and also has a wide range of applications in bio-nanotechnology.^176^

Biomimetic magnetosomes acting as multifunctional artificial antigen presenting cells (aAPCs) were developed by Zhang and team by coating azide-modified leucocyte membrane over magnetic nanoclusters through bio-orthogonal click chemistry. The aAPCs facilitate provocation and proliferation of cytotoxic T-cells and also assist in their reinfusion of cytotoxic T-cells into tumor tissue *in vivo*, aided by magnetic resonance imaging and magnetic control properties of aAPCs, inhibiting the development of the tissue.^177^

Accumulation of antibodies in the main organs of the body could induce cytotoxic side-reaction and also create difficulty in imaging of the system. Therefore, to overcome the problem of accumulation Smith *et al.* developed antidotes with clickable properties which effectively decline the level of antibodies in the circulatory system of the body. Nanoparticles were modified with *trans*-cyclooctyne and made to couple with the antibodies bearing methyltetrazine *in vivo*. The nanoantidote created with several set of appropriate drugs possessing short half-life and fast click kinetics eradicates the antibodies with long half-life.^178^

HIV, Human Immunodeficiency Virus, is a significant global health challenge. It attacks the immune system, specifically the CD4 cells (T cells), which help the immune system fight off infections. If left untreated, HIV can lead to Acquired Immunodeficiency Syndrome (AIDS), which is lethal to living beings. Ledezma and the team works to conquer the challenge and implement copper-free click chemistry wherein they conjugate analogues, azide and alkyne group, at the active-site of DNA polymerase suppressing the HIV reverse transcriptase activity and prevent its proliferation.^179^

Madl *et al.* developed elastin like proteins (SPAAC cross linked materials) to form gels within seconds and gelation in a couple of minutes. These gels support the *in vitro* culture of human mesenchymal stem cells, murine neural progenitor cells and human umbilical vein endothelial cells with approximately 97% viability and phenotypic maintenance for various tested cells. Matrix elasticity tuning, cell conjugate ligand and sequence of amino acids in bioactive molecules have no impact over the SPAAC crosslinked gels making them beneficial in bioprinting and also in therapeutic cell delivery.^180^

**Fig. 34:**
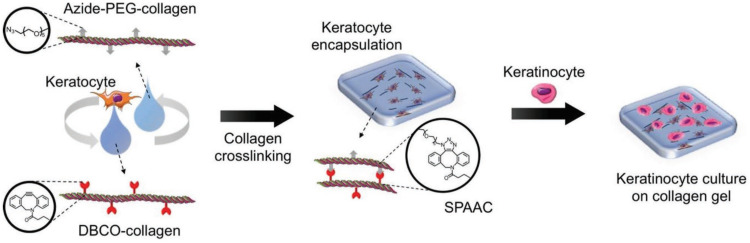
A crosslinked gel formation using strained promoted azide alkyne cycloaddition. Reproduced from ref. 181 with permission from John Wiley and Sons, copyright 2018.

Similar work was done by Lee and the group wherein they prepared corneal stromal substitutes derived from collagen type I using bio-orthogonal click chemistry. The properties of the gel were controlled by using specific ratios of functional groups and collagen concentration. Compared to non-crosslinked gels, the SPAAC crosslinked gels have more gel integrity providing ideal substrate stiffness and also withstanding decomposition. Fabrication of corneal tissue substitute was done by combining azide-PEG-conjugated collagen and DBCO-modified collagen with keratocytes^181^ as shown in the Fig. 34.

**Fig. 35:**
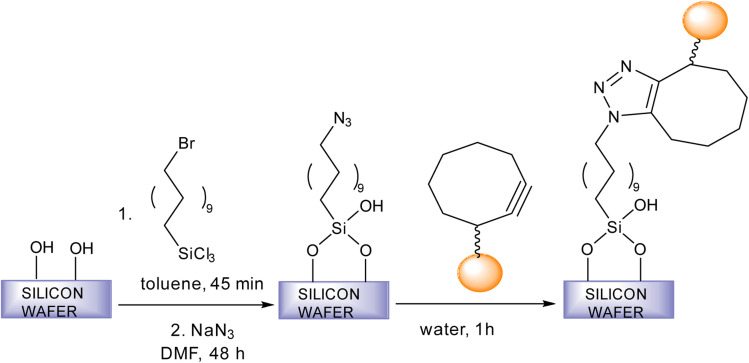
An alteration in magnetic nanoparticles through SPAAC.^182^ Reproduced from ref. 182 with permission from Royal Society of Chemistry, copyright 2017.

Fratila *et al.* synthesise physiologically stable magnetic nanoparticles using hydrophilic cyclo-octynyl-amine derivatives showing coupling reaction with azide-functionalised silicon substrates *via* SPAAC making the nanoparticles sedentary as shown in Fig. 35. For making the nanoparticles water and physiologically stable, the addition of polyethylene glycol or 4-aminophenyl β-d-glucopyranoside was proposed. The reactivity of the magnetic nanoparticles show variation with the functionality of the azide surface.^182^

**Fig. 36:**
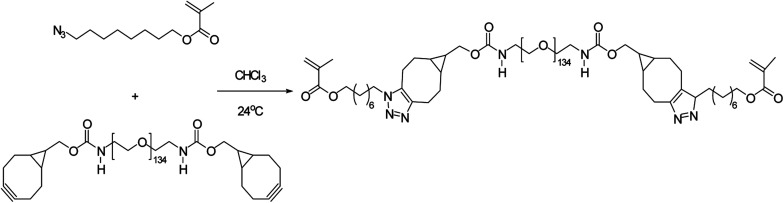
Development of bio-adhesive polymer through metal-free click chemistry.^183^ Reproduced from ref. 183 with permission from Royal Society of Chemistry, copyright 2018.

Poly(2-methacrylamido glucopyranose-*co-N*-methacryloyl-3,4-dihydroxyl-l-phenylalanine-*co*-8-azidooctyl methacrylate), a bio-adhesive based glucose was synthesised by Pramudya and team by exploiting metal-free click chemistry. Poly(MG-*co*-MDOPA-*co*-AOM) was covalently bonded to poly(ethylene glycol) (PEG)-based crosslinker through SPAAC as shown in Fig. 36. ^1^H NMR and FT-IR verified the instance of coupling through SPAAC and also the adhesion strength of the conjugated polymer was analysed through lap shear strength test over the skin of porcine which came out to be extremely significant. SPAAC with its biocompatible feature also gave an opportunity to control the bond length of terpolymer.^183^

**Fig. 37:**
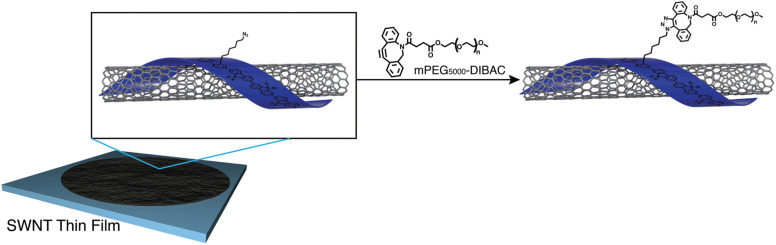
Modulation of carbon nanotubes through bio-orthogonal click chemistry. Reproduced from ref. 184 with permission from the Royal Society of Chemistry, copyright 2010.

Fong *et al.* have enhanced the properties of carbon nanotube, polymer-SWNT (single-walled carbon nanotubes), by exploiting strained promoted alkyne azide cycloaddition owing to the facts that the technique is biocompatible, site-specific and produces insignificant by-products. They incorporate azide group in the side chain of polyfluorene, utilized in the development of thin films nanotubes, and then conjugate it with cyclooctyne derivative of polyethylene glycol *via* SPAAC giving rise to 1,2,3-trizole ring at room temperature and without the aid of any catalyst as shown in Fig. 37. Analysis through Raman spectroscopy, fluorescence and absorption showed no alteration in the structural properties of the nanotubes. However, hydrophobicity was modulated accordingly.^184^

Qian *et al.* conducted an experiment to showcase the efficient production of potential caspase-1 inhibitors through the utilization of the copper-free strain-promoted alkyne–azide cycloaddition (SPAAC) reaction. The result was attained through the utilization of difluorinated cyclooctynes (DIFOs) in combination with diverse azide-containing compounds. The bicyclic compounds that were acquired displayed a structural similarity to the central region (P2–P3) of Pralnacasan, a well-known small molecule inhibitor of caspase-1. The incorporation of the azide component conveniently facilitated the introduction of diversity at the P4 position of the original inhibitor. The inhibitor library employed in our study was synthesized using copper-free bio-orthogonal chemistry techniques. The utilization of this approach facilitated the proficient construction of a library comprising 52 members, which encompassed 2 DIFOs and 26 azides. The library that was obtained was promptly prepared for screening using cells, with the aim of quickly identifying compounds that have the ability to enter cells and effectively inhibit the activities of endogenous caspase-1.^185^

**Fig. 38:**
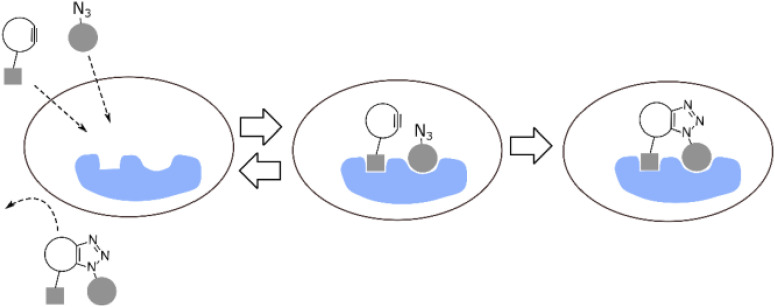
Formation of bioactive molecule using iSPAAC. Reproduced from ref. 186 with permission from John Wiley and Sons, copyright 2018.

The utilization of SPAAC for the synthesis of a bioactive compound, without the presence of isomers, was reported by Lis and colleagues in their research study. This technique was successfully employed both *in vitro* as well as *in vivo*. The researchers successfully synthesized the symmetrical cyclooctyne SYPCO and employed it to produce a chemically homogeneous triazole inhibitor for protein–protein interactions facilitated by Bcl-xL. This was achieved through the utilization of isomer-free strain-promoted azide–alkyne cycloaddition (iSPAAC) methodology as shown in Fig. 38. The tumor cells that were subjected to the reactants of the iSPAAC reaction exhibited elevated concentrations of triazole and demonstrated increased levels of apoptosis compared to the cells treated with pre-synthesized triazole. The iSPAAC method is envisioned to have wide applicability in the modulation of intracellular targets using organic molecules that have molecular weights that are too large for cellular uptake. This is achieved by utilizing smaller components that are more permeable to cells.^186^

Tissue adhesives were also reported by Li and the research group with an adhesion time of less than 2 minutes, making them ideal for surgical use. Usually, contemporary tissue adhesives are associated with cytotoxicity, ineffective adhesion strength, and high cost. However, the reported adhesives are more feasible and are synthesized through the conjugation of tetrazine in chitosan *via* copper-free click chemistry.^187^

### 5.6. Membrane fusion

Membrane fusion is a cellular process wherein lipid bilayers of two distinct membranes merge, allowing the mixing of their contents, and is facilitated by specialized proteins that bring the membranes into proximity and induce their structural rearrangement. This phenomenon is crucial for various biological events, such as vesicle trafficking, cell communication, and viral entry. Whitehead *et al.*, designed and synthesized ODIBO lipid which is relatively more efficient for membrane derivatization and triggering of fusion when compared with other ADIBO lipid derivatives, provided that the fusion in the physiological conditions takes place through SPAAC. The lipid part is incorporated in the unilamellar liposomes to examine fusinogenic properties.^188^

**Fig. 39:**
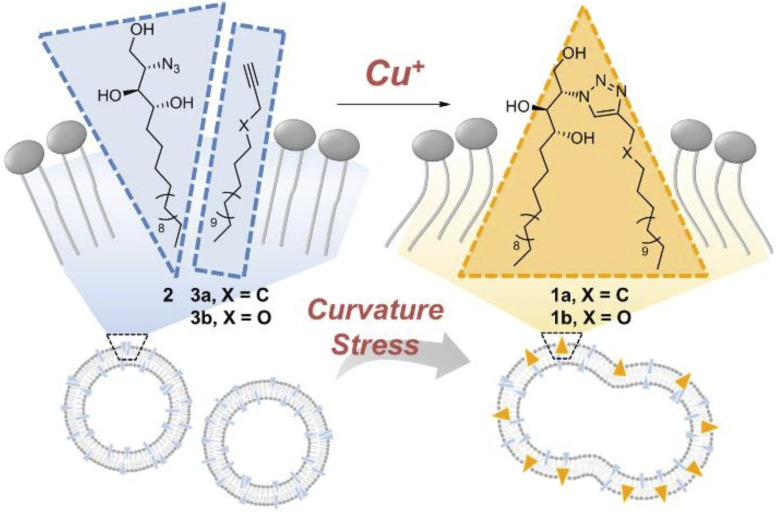
CuAAC click reaction enabling membrane fusion. Reproduced from ref. 189 with permission from John Wiley and Sons, copyright 2019.

Lee *et al.* unraveled the driving force for membrane fusion, focusing on ceramide's role. By crafting artificial membranes where only curvature changed, it was observed that the fusion was triggered solely by ceramide's shape-shifting act. A cone-shaped molecule modified with azido-group bent the membrane, thereby proving curvature. CuAAC ‘click’ reaction between the cone-shaped azido molecule and the terminal alkyne as liposome surface (as shown in Fig. 39), not only sheds light on cellular processes but also offers a cytocompatible platform for future biomedical applications ranging from drug delivery to tissue engineering.^189^

### 5.7. Polymerization

Metal-free thermal alkyne azide clickable polymerization can be used instead of metal-catalyzed one, where the reaction mixture is subjected to a thermal energy source in place of using metal as a catalyst, to synthesize sustainable polymer. One example of such polymerization was reported by Çakmakçı and his co-workers, wherein the synthesis of vegetable-oil-based antibacterial thermoset materials was done *via* click chemistry. Tetra-propargyl functionalized dimer diamine, prepared by Diels–Alder reaction, was coupled with azidated trimethylolpropane triglycidyl ether and castor oil, phosphorylated to enhance thermal properties of antibacterial thermosets, *via* Thermal Alkyne Azide Cycloaddition (TAAC) producing the thermoset material which was resistant to both Gram-positive and Gram-negative bacteria and the structure of the material was characterized by FTIR and NMR showing results corresponding to the elastomers.^44^

**Fig. 40:**
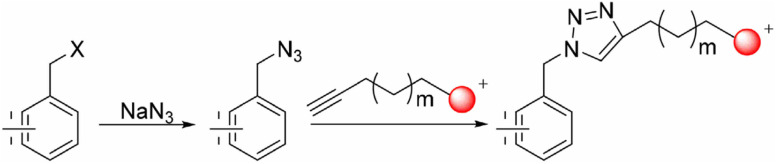
General scheme for the synthesis of AEM through CuAAC. Reproduced from ref. 190 with permission from John Wiley and Sons, copyright 2022.

Anion exchange membranes (AEMs) have been a cornerstone of energy and environmental technologies, serving as vital functional materials. Yang *et al.*^190^ studied click chemistry revolutionizing AEM synthesis with its efficiency, versatility, and safety. This innovative approach offers a clear advantage over traditional methods. Click reactions are delightfully simple, dramatically boosting the preparation efficiency of AEMs, granting a vast toolbox to conjugate terminal alkyne and azide groups (as shown in Fig. 40), unlocking a universe of AEM architectures and cationic species. The azide group has been introduced through NaN_3_ while the terminal alkyne has been incorporated by using poly (aryl ether) and also polystyrene mixed with flexible polymers synthesizing potent reactants for CuAAC click reaction.

**Fig. 41:**
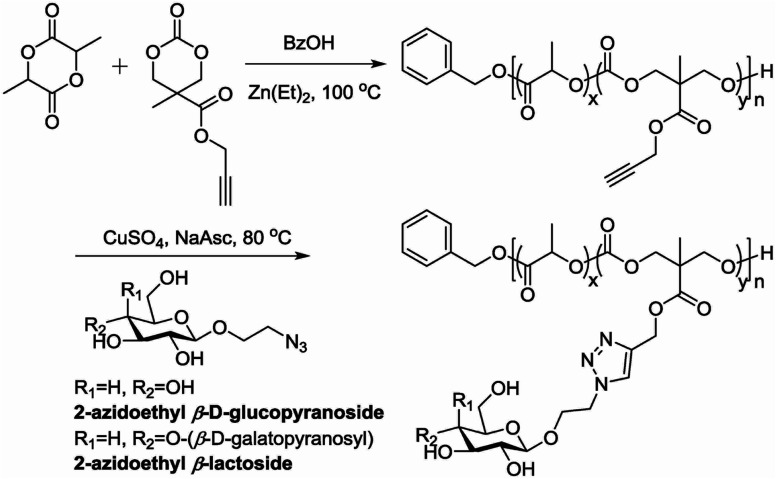
Polymerization of carbonate monomers through CuAAC click reaction. Reproduced from ref. 191 with permission from John Wiley and Sons, copyright 2017.

Dai and group analyzed click chemistry, which has revolutionized the design of functional aliphatic polycarbonates, a family of biodegradable polymers with promising applications in medicine and materials science. These polymers, characterized by their carbonate bonds, can be tailored with specific functionalities using click reactions. A cyclic carbonate monomer (MPC), allows the creation of biodegradable poly(l-lactide-*co*-carbonate) through co-polymerization. By incorporating clickable alkyne groups into poly(d-lactide-*co*-carbonate), it can be further modified with sugars like azidoethyl β-d-glucopyranoside and β-lactoside *via* click chemistry as shown in Fig. 41. This results in functional copolymers with unique binding affinity for lectin molecules, opening doors for targeted drug delivery and biosensing applications. These polymers' inherent biodegradability and biocompatibility make them prime candidates for medical applications, while click chemistry's success under physiological conditions further broadens their potential. Notably, click chemistry has already been used to create these polymers for polymeric micelles (drug carriers), prodrugs (controlled drug release), hydrogels (tissue engineering scaffolds), and more.^191^

### 5.8. Structural modification

Protein translation is a fundamental biological process that occurs in cells, where the information encoded in the DNA is used to synthesize proteins. Post-translation modification of the proteins enriches the biomolecule. However, the process is associated with abstruseness. Hartley *et al.* exploited bio-orthogonal click chemistry, due to its site-specific characteristic, to simplify the process. They developed TEM β-lactamase modified with *p*-azido-l-phenylalanine at the site of interest, later formed adduct with alkyne *via* SPAAC modulating the activity of biomolecule.^192^

Luo *et al.* provide a comprehensive account of the processes involved in the synthesis, fabrication, and characterization of water-soluble gold nanoparticles (AuNPs) measuring approximately 3 nm in diameter. The nanoparticles in question are equipped with strained-alkyne functional groups that are masked by cyclopropenone. These functional groups demonstrate the capacity to engage in interfacial strain-promoted alkyne–azide cycloaddition (I-SPAAC) with azides when exposed to UV-A light. The introduction of a strained-alkyne precursor onto gold nanoparticles (AuNPs) was achieved by the researchers through a direct ligand exchange process. Specifically, they utilized a thiol-modified cyclopropenone-masked dibenzocyclooctyne (photoDIBO) ligand for this purpose. The research demonstrated the prompt and effective responsiveness of DIBO-AuNPs upon exposure to various 1,3-dipoles, under mild reaction conditions. The experimental findings indicate that DIBO-AuNPs tend to undergo chemical modifications with complex azide-bearing molecules. The synthesis of a conjugate between the AZT drug and AuNPs served as an exemplification of this phenomenon^193^

## 6. Conclusion and future prospective

The convergence of bio-orthogonal chemistry and click chemistry represents a pivotal paradigm in chemical biology. The amalgamation of selective, bio-compatible click reactions with the principles of bio-orthogonality has led to the development of powerful tools for precise manipulation and exploration of biological systems. The implementation of bio-orthogonal click reactions, exemplified by the CuAAC and SPAAC methodologies, has demonstrated remarkable success in facilitating bioconjugation, live-cell imaging, and targeted drug delivery. As research in this field progresses, the refinement of existing bio-orthogonal click reactions, the exploration of novel chemistries, and the expansion of applications in proteomics and genomics are anticipated. This synergistic approach between chemistry and biology not only advances our fundamental understanding of biological systems but also holds immense promise for translational applications in diagnostics, drug development, and personalized medicine.

However, the limitation posed by the toxicity of copper in the CuAAC reaction for *in vivo* applications remains a challenge. Future research may focus on developing alternative catalytic systems that are biocompatible and can overcome this limitation, allowing for broader applications *in vivo*. This could involve exploring new metal catalysts or even non-metal-based catalytic systems. Modifications aimed at enhancing reaction rates, selectivity, and efficiency could further broaden the scope of click chemistry. The quest for more sustainable and environmentally friendly reaction conditions may also drive developments in this field. As the applications of bio-orthogonal click chemistry continue to diversify, the future holds exciting possibilities, with ongoing efforts focused on overcoming limitations, expanding application areas, and fine-tuning reaction conditions for enhanced performance in both *in vitro* and *in vivo* settings.

## Conflicts of interest

There are no conflicts of interest to declare.

## Supplementary Material
